# Study on the Machining Characteristics of SiCp/Al Under Pulsed Laser–Ultrasonic Vibration-Assisted Composite Turning

**DOI:** 10.3390/mi17101144

**Published:** 2026-09-30

**Authors:** Guangze Du, Yan Gu, Bin Fu, Xuesong Wang, Lingling Han, Jiaxin Zhao, Bingjin Yu, Minglei Wu

**Affiliations:** 1 Jilin Provincial Key Laboratory of Micro-Nano and Ultra-Precision Manufacturing, School of Mechatronic Engineering, Changchun University of Technology, Yan’an Ave 2055, Changchun 130012, China; du17549541473@163.com (G.D.); 17748242912@163.com (X.W.); 1202501006@stu.ccut.edu.cn (L.H.); 17853318982@163.com (J.Z.); yubingjinccut@163.com (B.Y.); 17644044940@163.com (M.W.); 2 Jilin Province Key Laboratory of International Science and Technology Cooperation for High Performance Manufacturing and Testing, School of Mechatronic Engineering, Changchun University of Technology, Yan’an Ave 2055, Changchun 130012, China

**Keywords:** pulsed laser–ultrasonic vibration-assisted turning, SiCp/Al composite material, surface integrity, cutting force, finite element simulation

## Abstract

Owing to their lightweight nature, low coefficient of thermal expansion, and strong wear resistance, aluminum-based silicon carbide composites serve as pivotal components in aerospace applications. Nonetheless, traditional cutting procedures face severe limitations. The abrasive resistance imparted by ultra-hard SiC particles induces severe load spikes and causes significant micro-damage during machining. To overcome these machining barriers, this study introduces a hybrid energy-field approach, namely, pulsed laser–ultrasonic vibration-assisted composite turning (LUAT). To deconstruct the thermo-mechanical coupling mechanism, a two-dimensional finite element simulation framework was constructed to investigate dynamic force responses and transient temperature fields. Leveraging a custom-built hybrid machining system, experimental investigations systematically evaluated dynamic cutting forces, areal roughness (Sa), and micro-scale damage mechanisms across conventional turning (CT), pulsed laser-assisted turning (PLAT), and LUAT processes. The findings confirmed that laser thermal softening lowered matrix deformation resistance, while the high-frequency intermittent contact mode of LUAT effectively minimized average cutting forces compared with all benchmark processes. Operating at 40 W laser power, 600 r/min rotational speed, 4 μm cutting depth, and 2 mm/min feed rate, LUAT achieved its lowest surface roughness of Sa = 0.075 μm, demonstrating a 56% improvement over laser-assisted turning alone. This work provides valuable theoretical and practical insights for advancing the high-efficiency precision manufacturing of hard-to-machine composites with high volume fractions.

## 1. Introduction

Owing to the combination of the lightweight and high-thermal-conductivity aluminum matrix and the high-hardness and low-coefficient thermal expansion of the SiC reinforcement phase, aluminum matrix silicon carbide (SiCp/Al) has been widely applied in cutting-edge fields including advanced weaponry, aircraft structural components, and precision electronic packaging [1,2,3,4], playing an irreplaceable role in the precision development of high-end equipment systems. To meet the stringent assembly requirements and harsh working conditions of precision components, the high-precision and high-surface-integrity machining of SiCp/Al composites must be achieved [5,6,7]. However, the multiphase, heterogeneous, and anisotropic properties of SiCp/Al render it a typical difficult-to-machine material [8,9,10]. During conventional cutting processes, the hard reinforcement phase is highly prone to inducing intense frictional thermo-mechanical load impact at the tool–workpiece contact interface [11,12,13]. Brittle fracture and fragmentation of reinforcement particles, interfacial debonding and spalling, and entire particle pull-out leave a large number of pits, cracks, and deep grooves on the machined surface, which significantly degrades surface quality [14,15,16,17]. With regard to the ductile metal matrix, the high temperature generated during cutting induces high viscoplastic flow of the aluminum matrix and the formation of built-up edge, which further exacerbates the deterioration of machined surface quality [18,19,20]. Therefore, it is imperative to develop a new precision machining technology that can effectively weaken the interference of hard particles, inhibit matrix adhesion, and obtain high-integrity machined surfaces.

Applying external energy fields during material removal has emerged as a prominent research direction for enhancing composite machinability [21,22,23]. Numerous studies have confirmed that energy-field assistance—spanning laser, ultrasonic, and hybrid laser–ultrasonic modes—effectively alleviates mechanical loads and lowers cutting resistance [24]. Among these, pulsed laser-assisted turning (PLAT) utilizes focused thermal energy from high-power laser beams to locally heat the workpiece, inducing thermal softening. While thermal softening weakens matrix shear resistance and promotes ductile removal of hard particles, excessive energy flux can induce thermal defects—such as microcracking and heat-affected layers—in the sensitive aluminum matrix [25,26]. Conversely, ultrasonic vibration-assisted turning (UVAT) applies high-frequency, micro-scale tool oscillations to establish a discontinuous contact interface, enhancing cutting stability and lowering mean force levels [27,28]. Consequently, LUAT was developed to synthesize laser-induced thermal weakening with ultrasonic intermittent contact dynamics. Under LUAT, pulsed laser irradiation first conditions target regions of the hard composite into a softened state, after which high-frequency ultrasonic separation dynamics drive material removal. This combined approach lowers mechanical cutting loads while expanding chip evacuation clearance, which boosts heat dissipation, enhances chip clearance, and mitigates PLAT-induced thermal damage.

Due to the extremely complex multiphase removal mechanism during the cutting process of composite materials, the traditional trial-and-error experimental method not only consumes substantial human and material resources but also makes it difficult to conduct direct dynamic acquisition of core physical quantities such as transient stress field and strain rate in the cutting area, which limits the research efficiency of the material removal mechanism [29]. In recent years, the finite element method (FEM) has been widely applied to analyze the microscopic interaction between cutting tools and heterogeneous particles, owing to its capability to effectively predict experimental results and obtain intermediate multiphase interaction information [30,31]. Based on the cohesive zone model (CZM) or macro–micro-multiphase random distribution reconstruction technology, some scholars have effectively simulated the brittle fracture, debonding and spalling processes of heterogeneous particles under different relative motion trajectories, including on, above and below the cutting path, and successfully predicted the evolution trends of cutting force, cutting temperature and machined surface morphology [32,33,34,35]. However, most existing simulation methods focus on the motion function loading of traditional continuous mechanical cutting or single purely mechanical ultrasonic vibration. When external energy fields are introduced, the dynamic interaction response model of multiphase matrix under complex thermo-mechanical thermal softening gradient remains relatively deficient, which makes it difficult to fully characterize the removal mechanism under multi-field coupling [36,37]. For SiCp/Al, a typical multiphase difficult-to-machine material with high volume fraction and uneven particle size distribution, the multiphase material removal mechanism and dynamic damage evolution pattern under the nonlinear synergy of multiple energy fields have not been fully elucidated; meanwhile, systematic research on the reasonable process matching that achieves both low surface roughness and micro-damage suppression is still lacking.

In view of the above problems, this study first established an analytical model of cutting force under a composite energy field and deconstructed the multiphase material removal mechanism, subsurface damage morphology and the influence of laser power on the evolution of the temperature field during the cutting process by the two-dimensional microscopic multiphase finite element simulation method. On this basis, a composite machining experimental platform was built, and comparative experiments of CT, PLAT and LUAT were carried out. The feasibility of the LUAT machining of SiCp/Al composites was verified by in-depth analysis of cutting mechanical properties, SiCp/Al macro- and micro-morphology and machined surface integrity.

## 2. Materials and Methods

### 2.1. Experimental Materials

**Figure 1:**
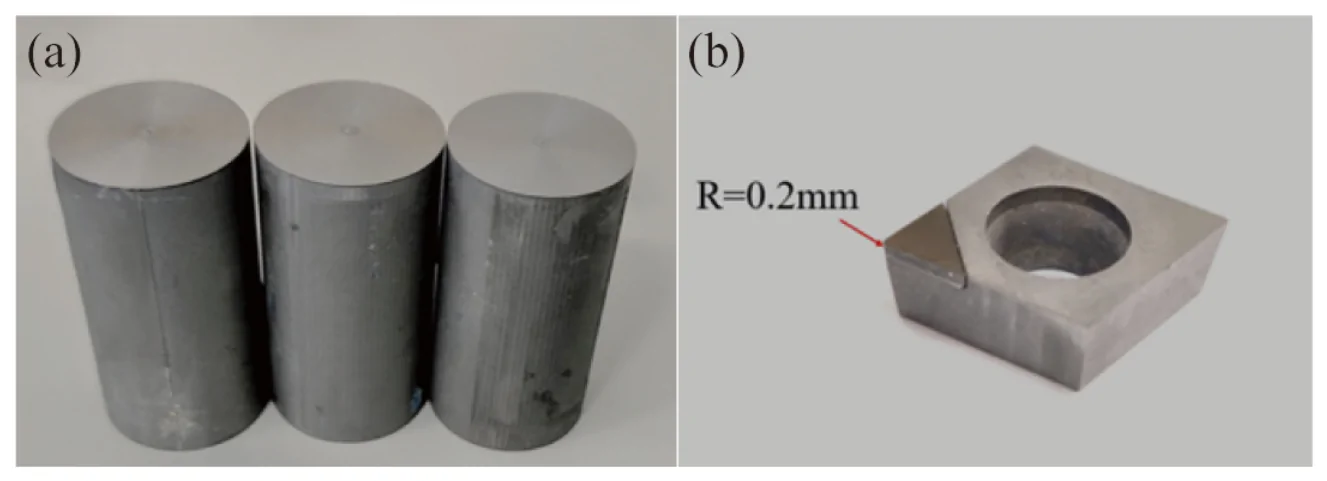
(**a**) 45%SiCp/Al. (**b**) PCD cutting tool.

A SiCp/Al composite featuring a 45% particle volume fraction and an average SiC grain diameter of approximately 30 μm was chosen as the test material. Xi’an Kurt New Materials Co. Ltd. (Xian, China). provided, and the manufacturing method was powder metallurgy. As illustrated in Figure 1a, cylindrical SiCp/Al specimens with dimensions of 25 mm in diameter and 50 mm in length were utilized. Rapid tool degradation frequently occurs when machining SiCp/Al composites owing to intense abrasive action from the hard SiC phase. To withstand severe wear, polycrystalline diamond (PCD) tools were adopted for the machining experiments on account of their superior hardness (Figure 1b). The specific parameters are presented in Table 1.

### 2.2. Working Principle of LUAT

**Figure 2:**
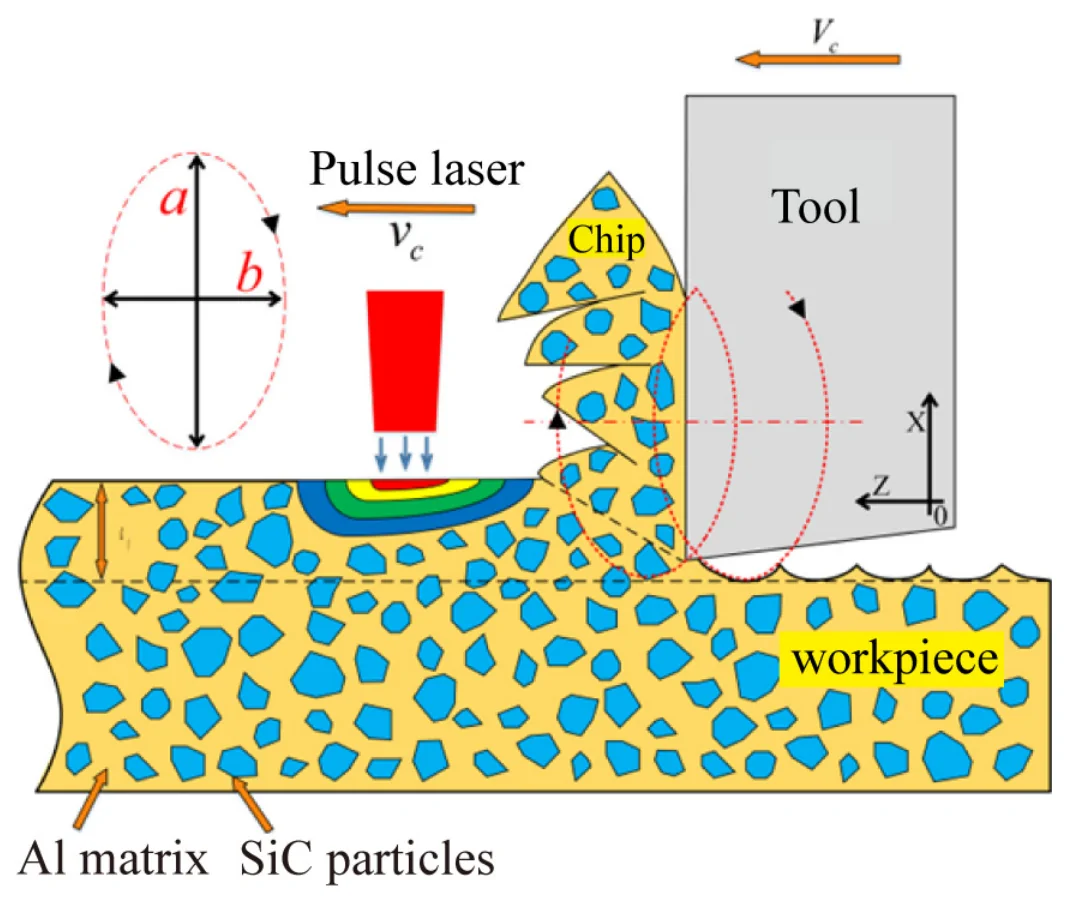
Principle of pulsed laser–ultrasonic vibration-assisted cutting.

The operational scheme of LUAT is depicted in Figure 2. By coupling a laser thermal energy field with an ultrasonic kinetic energy field, this hybrid technique enhances material removal. During machining, the laser spot remains focused on the unmachined surface, maintaining a constant separation relative to the synchronously advancing tool tip, while high-frequency ultrasonic vibration is applied directly along the cutting path. Upon exposure to the pulsed laser heat source, a sharp temperature rise occurs locally, inducing a thermal softening phenomenon that lowers the yield limit and deformation resistance of the SiCp/Al material. Kinematically, the ultrasonic excitation drives the tool tip to establish periodic, high-frequency intermittent contact with the workpiece substrate and internal hard phases. The high-frequency separation feature alters the tool–chip friction regime, reduces interfacial contact area, and facilitates microcrack propagation and chip fracturing through dynamic impacts. Consequently, the synergistic combination of thermal relaxation, acoustic impact, and friction alleviation under the hybrid energy fields yields a notable reduction in the overall average cutting force.

The velocity component in the cutting direction, $v_{t}\left(t\right)$, is given by the following expression:

(1) $$v_{t}\left(t\right)=2\pi fA_{x}\cos\left(2\pi ft+\varphi \right)$$

Here, f represents the ultrasonic frequency, and $A_{x}$ denotes the vibration amplitude along the cutting direction. The peak cutting edge speed of the tool along the cutting direction is $v_{t,max}=2\pi fA_{x}$.

## 3. Finite Element Simulation

Owing to the extremely short duration (on the microsecond scale) of the thermal effect of pulsed lasers and the mechanical impulse impact of ultrasonic vibration and given the fact that the cutting zone is in a closed state, conventional physical experimental methods such as cutting force measurement can only reflect macroscopic responses but are unable to directly conduct in situ observation of the dynamic evolution process of micro-scale stress distribution, crack propagation, and multiphase interface damage. Accordingly, this section establishes a two-dimensional micro-scale finite element model (FEM) to investigate the cutting response of SiCp/Al composites under thermo-mechanical coupled loads. A comparative analysis is conducted on the micro-scale material removal behavior, cutting heat and stress-field evolution mechanisms of the composites under three cutting processes: CT, PLAT, and LUAT.

### 3.1. Simulation Setup

Using Python 3.12.0-based secondary development in Abaqus, a custom script was written to generate random polygonal particles and construct a two-dimensional microscopic multiphase finite element model of the 45% SiCp/Al composite. To effectively visualize material removal dynamics and particle failure behaviors, the two-dimensional workpiece domain was dimensioned as 0.6 × 0.3 mm. Reinforcement particles were constrained within a 15–35 μm size range, maintaining a fixed 45% volume fraction with spatial randomness across the aluminum matrix. To align with experimental conditions, the tool geometry was specified with a 0° rake angle and a 7° clearance angle. Owing to the superior hardness of PCD relative to the 45% SiCp/Al composite, tool compliance and abrasive wear were omitted, modeling the cutter as a non-deformable analytical rigid body. Table 2 summarizes the specific parameter settings adopted for the finite element simulation.

**Figure 3:**
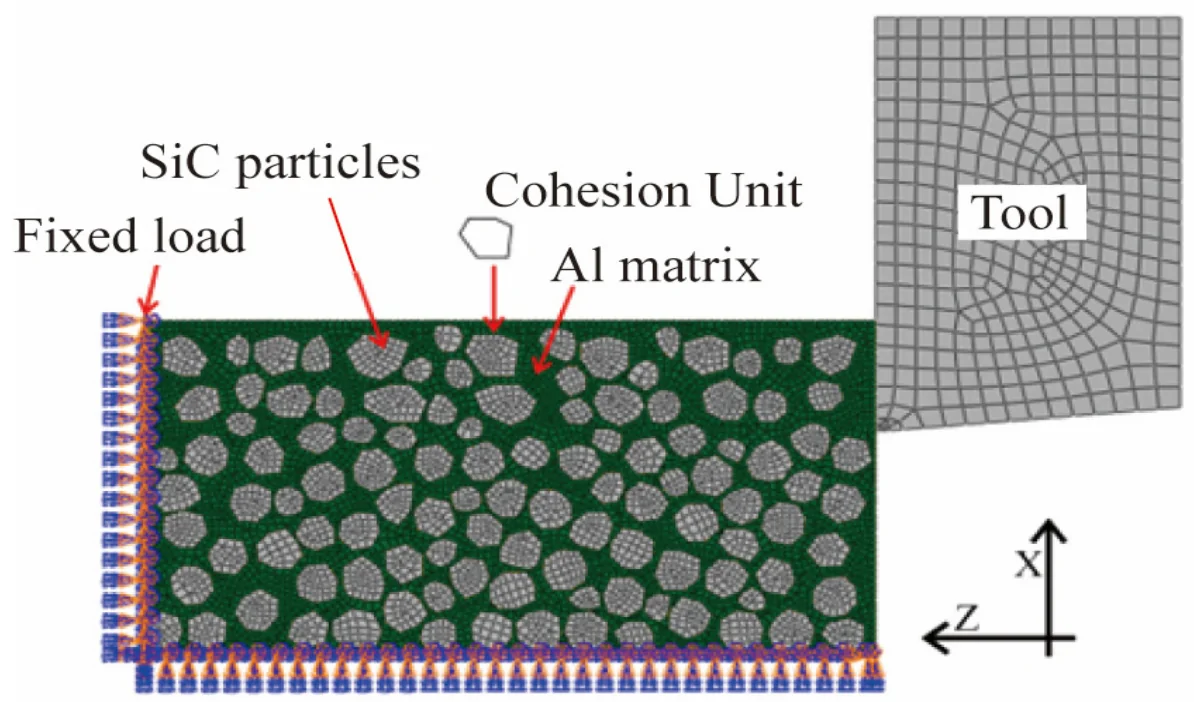
Two-dimensional finite element cutting model.

The two-dimensional finite element cutting model of 45% SiCp/Al is shown in Figure 3. The tool–workpiece interaction relationship is established through part assembly and contact property definition. In the configuration of contact behavior, in addition to defining the master–slave contact between the workpiece and the tool surface, the self-contact property is introduced into the workpiece to prevent mesh penetration and solid self-intersection during chip formation under high-strain-rate conditions. In terms of boundary condition configuration, fully fixed constraints are imposed on the left side and bottom surface of the workpiece. In the meshing stage, to optimize computational efficiency while ensuring calculation accuracy, the average global mesh size is set to 6 μm. For the two-phase interaction between SiC particles and the Al matrix, in addition to conventional contact pairing, a layer of zero-thickness cohesive elements (cohesive zone model, CZM) is embedded at the two-phase interface as the interphase, to accurately simulate the interfacial bonding strength between particles and the matrix.

The Johnson–Cook (J-C) model is selected to describe the dynamic behavior of the Al matrix during the machining process, and the J-C constitutive model is presented as Equation (2).

(2) $$\sigma (\varepsilon_{p},\varepsilon ,T)=(A+B\varepsilon_{p}^{n})\left[1+Cln\left(\frac{\dot{\varepsilon }}{{\dot{\varepsilon }}_{0}}\right)\right]\left[1-{\left(\frac{T-T_{r}}{T_{m}-T_{r}}\right)}^{m}\right]$$

In the equation, A denotes the initial yield stress of the material; B is the strain hardening parameter; C is the strain rate strengthening coefficient; *ε* is the equivalent plastic strain; *n* is the hardening index; $\dot{\varepsilon }$ is the equivalent plastic strain rate; ${\dot{\varepsilon }}_{0}$ is the reference strain rate; T_r_ is room temperature, which is set to 23 °C; T_m_ is the melting temperature of Al; and m is the thermal softening exponent of the material.

In the model, the J-C failure criterion is employed to represent the initial damage of the Al matrix material:

(3) $$e^{f}=\left[D_{1}+D_{2}exp\left(D_{3}\frac{P}{\sigma }\right)\right]\left(1+D_{4}ln\frac{\dot{\varepsilon }}{{\dot{\varepsilon }}_{0}}\right)\left(1+D_{5}\frac{T-T_{r}}{T_{m}-T_{r}}\right)$$

In the formula, e^f^ represents the critical equivalent plastic strain, while D_1_, D_2_, D_3_, D_4_, and D_5_ are material failure parameters, and P is the static water pressure. The material parameters of 6005Al and the J-C constitutive parameters in the model are provided in Table 3. The temperature field distribution was calculated based on a moving Gaussian heat source model (see Supplementary Materials for detailed modeling procedures).

### 3.2. Simulation Characteristics of Dynamic Cutting Force

**Figure 4:**
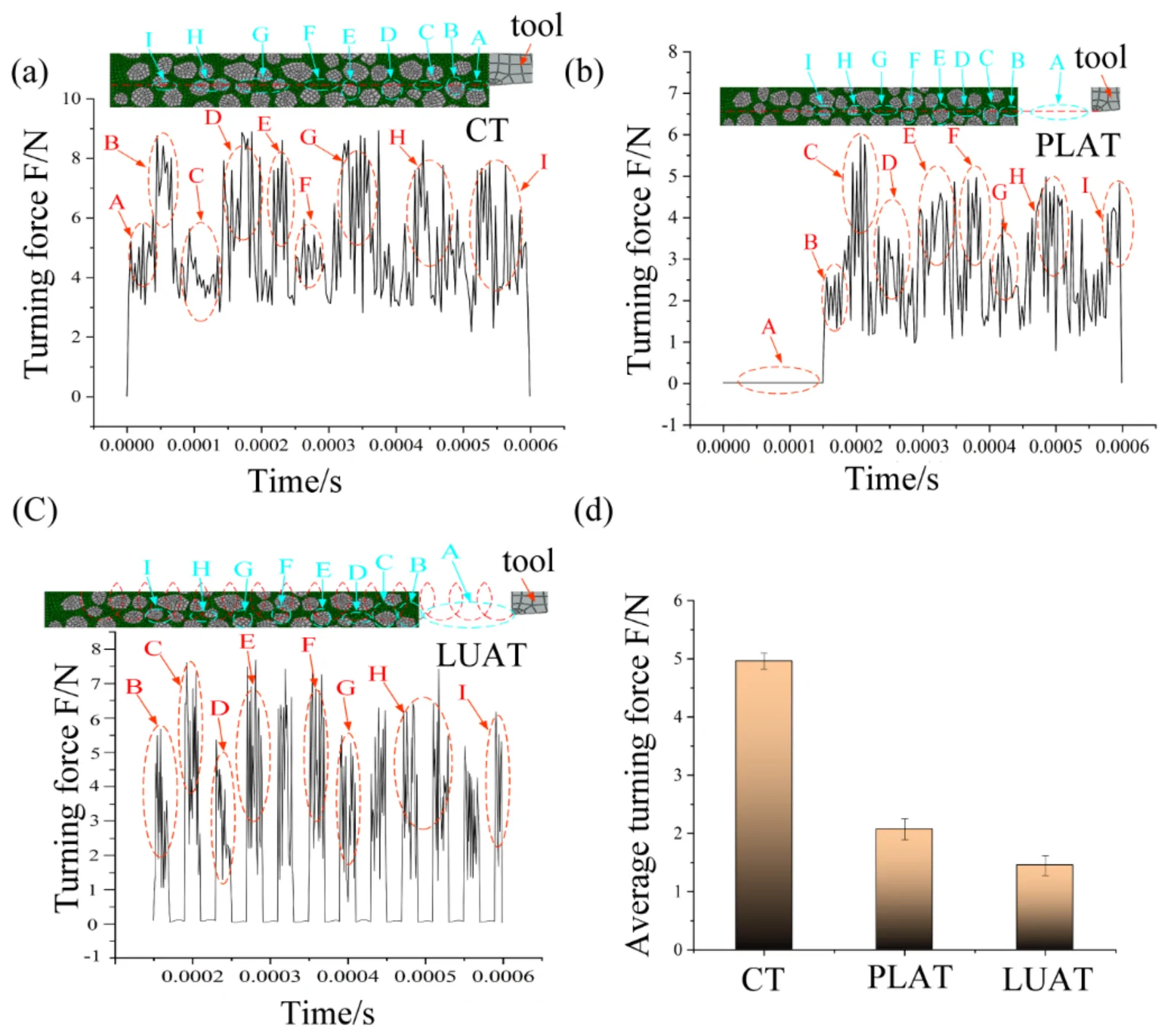
(**a**) Simulated cutting force profile for CT. (**b**) Simulated cutting force profile for PLAT. (**c**) Simulated cutting force profile for LUAT. (**d**) Comparison of mean cutting forces across the evalu-ated processes.

As a core physical quantity characterizing the cutting state, cutting force directly reflects the interaction mode between the cutting tool and composite materials. Figure 4 presents the finite element simulation curves of cutting force varying with time and the comparison of average cutting forces under different processing methods.

Figure 4a illustrates the time-history curve of cutting force obtained via finite element simulation under CT, with the contact position between the tool and workpiece and the tool motion trajectory visually displayed in the upper part of the figure. At the initial stage, when the tool cuts into the workpiece, the cutting force increases instantaneously, then maintains narrow-range fluctuations during the stable material removal phase of the matrix, with an average cutting force of approximately 4.3 N (as shown in Area A in the figure). As cutting progresses, the tool contacts SiC particles on the cutting path. Since the hardness of SiC particles is far higher than that of the Al matrix, severe load impact is induced at the instant of tool–particle contact, causing the cutting force to jump to a peak value of approximately 8.4 N (as shown in Area B), at which point the particles fracture and break under the extrusion of the tool. When processing enters Area C, part of the broken particles are discharged with chips, while the remaining part adheres to the machined surface. The tool separates from the particles, leading the cutting force to drop back and fluctuate within the range of a 5.7 N peak. As the cutting stroke continues, the tool cuts into the particle-intensive area again (as shown in Area D in the figure). Within this area, the high-density particles not only fracture and debond themselves under the tool’s pushing but also generate intense micro-collisions with adjacent particles. This inter-particle chain collision further exacerbates the dynamic fluctuation of cutting force, pushing the peak cutting force in this area to 8.6 N. Eventually, cutting ends in Area I, and the cutting force gradually decreases to zero. In conclusion, the cutting force in the CT process exhibits strong non-stationary fluctuation characteristics, which is mainly caused by the alternating interaction between unevenly distributed SiC particles and the Al matrix, as well as the dynamic change in the relative position between the tool and particles; meanwhile, the obstruction of hard particles is the core factor inducing high-amplitude impact force.

Figure 4b shows the finite element simulation cutting force curve under PLAT. Different from CT, to simulate the preheating effect of laser in PLAT simulation, the initial position of the tool is set at a certain distance from the starting end of the workpiece when establishing the geometric model, while the laser spot remains focused on the front end of the workpiece. Therefore, in the initial stage (0~150 μs), the tool has not yet cut into the material, and the cutting force remains zero (as shown in Area A). When the tool cuts into the preheated material (Area B), the cutting force rises rapidly. Benefiting from the homogenization evolution of material properties, the fluctuation amplitude of cutting force is small at this stage, with an average value of only approximately 2 N. When the tool encounters SiC particles inside the workpiece (Area C), the cutting force undergoes a sudden peak mutation again (approximately 6.1 N), accompanied by intensified fluctuation. As cutting continues to advance into the densely particle-distributed Areas E and F, characteristics different from CT are observed: the peak cutting force in this high-density area (approximately 5 N) does not increase further but is lower than that in Area C. This is mainly attributed to the thermal softening effect induced by laser irradiation: as processing time increases, the accumulated laser thermal energy inside the workpiece increases continuously, and the thermal softening effect becomes increasingly significant, leading to a substantial reduction in the plastic flow stress and tensile strength at the interface between the Al matrix and SiC particles. Therefore, the overall cutting force under PLAT mode is significantly lower than that under CT, and the peak cutting force shows a gradually decreasing trend along with the cutting process. Eventually, cutting ends in Area I, and the cutting force drops to zero.

Figure 4c depicts the simulated dynamic cutting force evolution during LUAT, highlighting the thermo-mechanical synergy between laser-induced softening and ultrasonic kinematics. In the Region A preheating stage, micro-inertial oscillations hover near zero. Upon tool engagement at 150 μs in Region B, the cutting force surges to an initial peak of 5.6 N. Instead of suffering severe thermal adhesion under sustained contact, the ultrasonic intermittent separation drastically narrows the effective contact duty cycle, limiting interface heat accumulation and maintaining low average engagement resistance. As the tool encounters hard SiC particles in Region C, the cutting load rises to a 7.6 N peak. Crucially, the hybrid fields avert catastrophic particle debonding: while laser softening grants localized compliant yield in the neighboring Al matrix, ultrasonic high-strain-rate pulses concentrate stress within the SiC phase, favoring localized micro-fracture over bulk particle pull-out. This suppresses violent force spikes and minimizes flank plowing wear. In the steady cutting regime in Region D, the dynamic force settles into an asymmetric sawtooth-profile rapid rise and gradual decay with a shortened action window and a reduced 5.4 N peak. This decay embodies the coupled synergy: the instantaneous cutting velocity exceeding the feed speed generates a steep impact peak, which is immediately balanced as cyclic deceleration combines with the heightened thermoplastic flow of the heated Al matrix. Consequently, LUAT avoids the sustained high-load regime of conventional cutting, optimizing the dynamic stress environment of the tool.

A comparative summary of average cutting forces across the three machining techniques is presented in Figure 4d. CT generated the highest average cutting resistance, recording approximately 4.9 N. Integrating pulsed laser irradiation induced a strong thermal softening effect, which lowered material deformation resistance and decreased the average cutting force to 2.1 N. In LUAT mode, despite higher transient peak forces and fluctuation amplitudes driven by high-frequency acoustic impacts, the periodic tool–workpiece separation feature decreased the duty cycle of active cutting. Consequently, LUAT achieved the lowest overall mean cutting force at just 1.5 N.

### 3.3. Transient Cutting Temperature-Field Change

During the cutting process, the mechanical heat generated by plastic shear and tool–workpiece interface friction, in conjunction with the high-energy heat source applied by pulsed laser, collectively drives the complex transient temperature-field evolution in the cutting zone. As the core link connecting multi-energy-field input and material mechanical response, the spatial evolution of the temperature field directly determines the thermal softening degree of the workpiece material, which in turn affects the stress-field distribution and cutting force evolution analyzed in the previous chapter. Meanwhile, the high-frequency separation effect of ultrasonic vibration alters the heat input and heat dissipation conditions in the cutting zone. Therefore, clarifying the evolution law of the transient temperature field under the three processing conditions can not only reveal the synergistic mechanism of laser thermal softening and ultrasonic heat dissipation but also lay a thermodynamic foundation for the subsequent analysis of micro-damage removal and macroscopic surface integrity.

**Figure 5:**
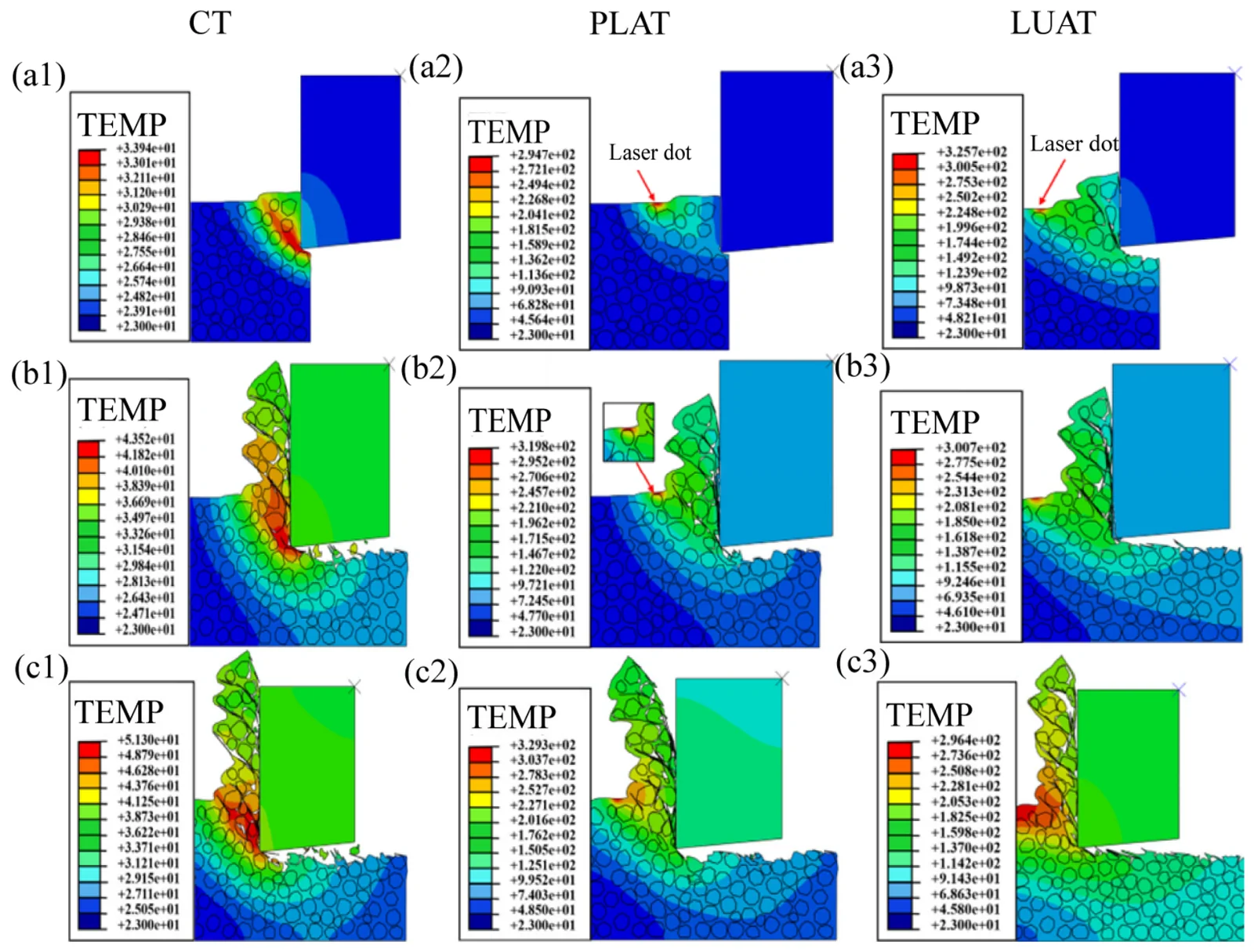
Temperature field distributions in the cutting zone under different machining methods at various cutting times: CT (**a1**–**a3**) 24 μs; PLAT (**b1**–**b3**) 150 μs; LUAT (**c1**–**c3**) 294 μs.

When the cutting time of the CT method reaches 24 μs, the tool has just begun to contact the workpiece. Due to shearing, the workpiece generates a relatively high temperature near the shear band. Moreover, owing to the friction between the tool rake face and the workpiece, the temperature of the tool–chip contact area also rises and is conducted toward the tool, as shown in Figure 5(a1). As cutting proceeds, when the cutting time reaches 150 μs, the tool comes into contact with SiC particles. Since SiC particles possess high hardness, the shearing interaction between the tool and the workpiece is enhanced at this stage. Under enhanced shearing and friction, the internal temperature of the workpiece rises rapidly and continues to be conducted toward the interior of the workpiece, as shown in Figure 5(b1). When the cutting time reaches 294 μs, the internal temperature of the workpiece continues to rise under the effect of heat conduction, with the maximum temperature occurring at the internal shear band of the workpiece. At this time, due to chip evacuation, the temperature inside the initially formed chips gradually decreases under the effects of heat convection and heat radiation. In addition, since partial regions of the workpiece are far from the shear band and cannot receive timely heat conduction, room-temperature regions form inside the workpiece, as shown in Figure 5(c1).

For cutting processing, pulsed laser irradiation induces a high-temperature gradient on the workpiece surface, while the temperature generated by cutting shearing is relatively low. Therefore, the temperature at the shear band is not prominent during the PLAT process, as shown in Figure 5(a2). As cutting time increases, the workpiece gradually conducts temperature from the surface to the interior under continuous pulsed laser irradiation, and the tool temperature also rises gradually after the tool contacts the workpiece. During cutting, when SiC particles are distributed near the workpiece surface, obvious changes in the temperature gradient near the particles can be observed due to the difference in thermal conductivity between the Al matrix and SiC particles, as shown in Figure 5(b2). This phenomenon mainly occurs because when heat is conducted from the high-temperature Al matrix to the vicinity of SiC particles, the thermal conductivity of SiC particles is lower than that of the Al matrix, allowing heat to transfer rapidly within the Al matrix. Consequently, the temperature inside SiC particles is relatively low, while the temperature of the Al matrix near SiC particles is relatively high. As cutting continues, the internal temperature of the workpiece and the tool temperature gradually accumulate and increase. Although the workpiece temperature near the surface is dissipated via heat convection and heat radiation, the temperature drop of the workpiece is not significant owing to the high temperature introduced by the pulsed laser, as shown in Figure 5(c2).

In the LUAT process, after the tool contacts the workpiece, it can be observed that the temperature in the shear zone is significantly higher compared with that in the PLAT process, as shown in Figure 5(a3). This is mainly because, compared with PLAT, under ultrasonic vibration, the tool achieves higher instantaneous cutting speed, which enhances the shearing interaction between the tool and the workpiece, thereby resulting in higher shear temperature. As the cutting distance increases, when the tool extrudes the workpiece, the friction between the tool rake face and the workpiece is enhanced, leading to an instantaneous increase in cutting temperature. Although ultrasonic vibration enhances the instantaneous shearing effect, within one elliptical vibration cycle, the tool’s vibration trajectory moves along the chip outflow direction. Under the effect of tool vibration, chip evacuation is facilitated, and the evacuated chips carry away a large amount of heat, which reduces the temperature of the workpiece, as shown in Figure 5(b3). Meanwhile, under ultrasonic vibration, the tool rake face separates from the chip, and the temperature of the separated tool is conducted from the tool tip to the interior of the tool; thus the tool tip temperature becomes lower than that of the chip. With intermittent cutting by the tool, most of the heat is carried away by chips and the tool, so the temperature near the workpiece shear band decreases relatively, while the temperature inside the workpiece gradually rises under the effect of heat conduction, as shown in Figure 5(c3).

### 3.4. FEA Deconstruction of Machined Surface Topography

The high-frequency fluctuation of mechanical shear load and the transient temperature-field gradient induced by pulsed laser act jointly, which alters the stress distribution of the aluminum matrix and the degree of stress concentration at the SiC particle interface in the cutting zone. This thermo-mechanical coupling effect directly dominates the micro-removal mechanism of SiCp/Al composites; that is, it determines whether the material tends to undergo brittle fracture of hard particles or high-plastic rheological encapsulation of softened matrix. Therefore, deconstructing the micro-removal behavior and surface topography during the cutting process via a two-dimensional finite element model (FEA) can clearly establish the mapping relationship of the evolution from the thermo-mechanical coupling field to material damage and removal.

**Figure 6:**
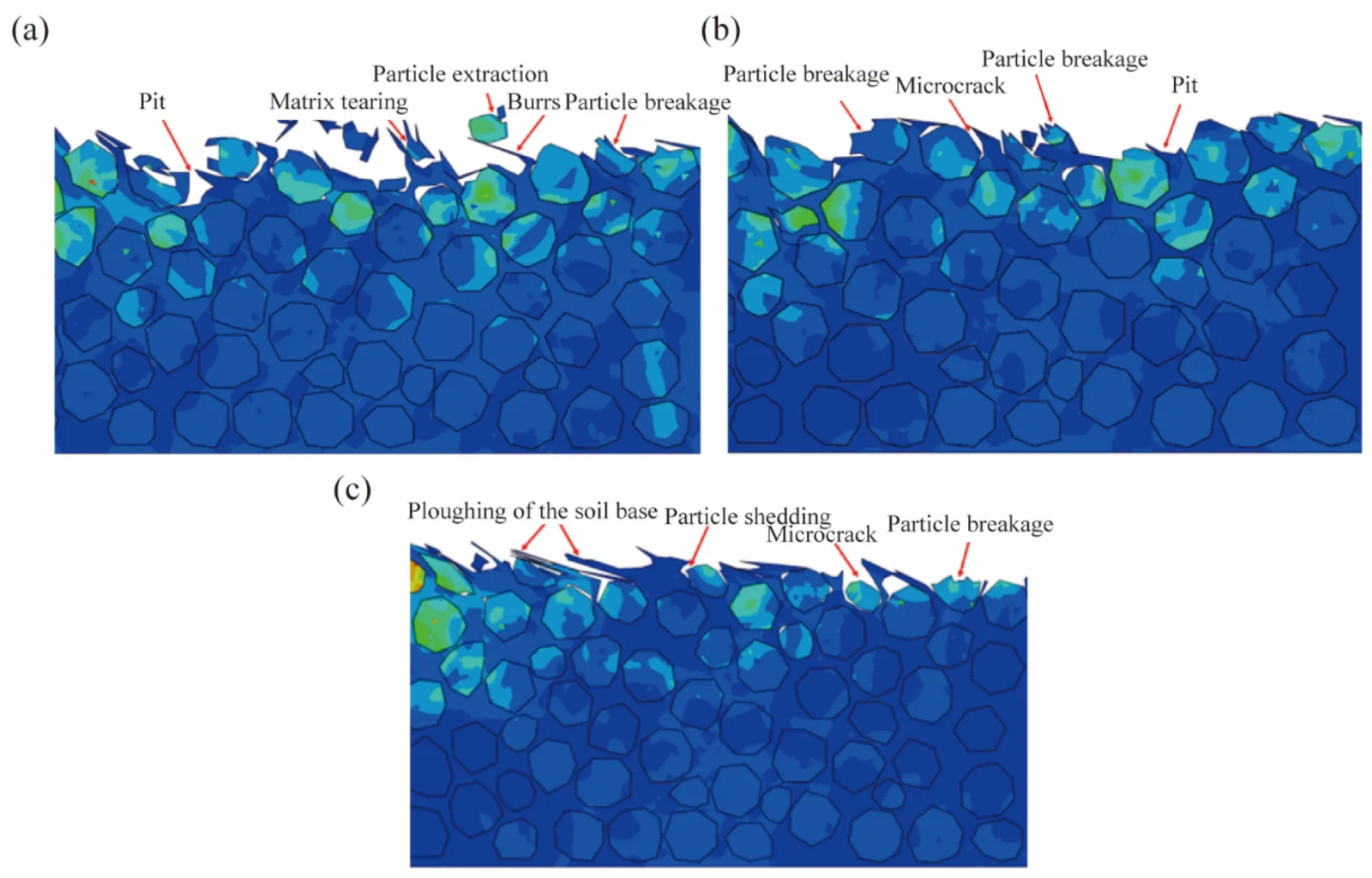
Surface morphologies during processing: (**a**) CT; (**b**) PLAT; (**c**) LUAT.

Figure 6a presents the FEA-based surface topography obtained under CT. Numerous fragmented particles are observed on the machined workpiece surface, and the Al matrix experiences tearing under tool action, resulting in extensive burr formation. Matrix tearing and SiC particle pull-out collectively lead to the deterioration of machined surface quality. This phenomenon is primarily attributed to the intense shear loading that particles on the tool’s cutting trajectory undergo, which induces significant stress concentration and consequently makes particles highly prone to fracture. Apart from a portion of fractured particles that are discharged along with chips, the remaining particles spall onto the workpiece surface, thereby altering the surface topography. When the tool acts on the lower portion of a particle, the particle is pulled out to form a chip under shear force, leaving a distinct pit on the machined surface. When the tool acts on the upper portion of a particle, if the stress generated at the tool–particle contact is lower than the interfacial debonding strength, the particle is not prone to debonding and tends to fracture at the upper portion or be pressed into the matrix by the tool. Conversely, if the interfacial bonding strength is lower than the maximum stress induced by tool shearing, the particle undergoes debonding, and the plowing effect is exerted on the interior of the workpiece under the pushing and extrusion of the tool, forming pit defects. Additionally, the non-uniform particle distribution in the composite material leads to poor tool stability and drastic cutting force fluctuations during CT, which is another critical reason for the high susceptibility to burr formation on the Al matrix under tool action.

The finite element analysis of the surface morphology of the workpiece processed by PLAT is shown in Figure 6b. The introduction of pulsed laser has an impact on the surface quality of the workpiece. Compared with conventional cutting (CT), the burrs on the surface of PLAT-processed specimens are significantly reduced, and the particle crushing phenomenon is remarkably improved. However, obvious pits, fracture of partial SiC particles and microcracks can still be observed. This is mainly attributed to the thermal softening effect induced by pulsed laser irradiation, which enhances the plasticity of the interior of SiCp/Al composites. When the tool interacts with the workpiece, although stress concentration still exists in the SiC particles along the cutting path, the highly plastic Al matrix wraps the SiC particles and moves synergistically under the extrusion of the tool, thereby alleviating the stress concentration of the particles and reducing their fracture tendency. In addition, the thermal softening effect reduces the hardness of the workpiece, which decreases the fluctuation of instantaneous cutting force when the tool contacts with the particles and the matrix, thus inhibiting the formation of burrs.

Figure 6c presents the FEA-based surface topography of LUAT-processed material. Compared with CT and PLAT, the surface machined by LUAT is smoother and flatter, no large-size pits are observed, and particle damage is mainly characterized by micro-fracture. The introduction of pulsed laser increases the plasticity of the workpiece and mitigates stress concentration during the cutting process, thereby inhibiting the severe fracture and debonding of particles. Meanwhile, ultrasonic vibration increases the instantaneous cutting speed of the tool. When the tool contacts a particle, the high strain rate makes the particle more prone to fracture. After fracture, a portion of the particles remain and are embedded smoothly in the machined surface, which avoids the formation of spalling pits and improves surface quality; the other portion is discharged in a timely manner under the high-frequency vibration of the tool. The synergistic mechanism of pulsed laser irradiation and ultrasonic vibration reduces the macroscopic hardness of SiCp/Al composites and collaboratively promotes the timely discharge of chips, resulting in superior surface quality compared with CT and PLAT.

In summary, the formulated two-dimensional finite element model (FEA) clarifies micro-scale material removal dynamics and damage evolution mechanisms in SiCp/Al composites under energy-field assistance through transient thermal conduction and dynamic mechanical perspectives. To examine how energy-field parameter variations affect macroscopic cutting forces, thermal distribution, and surface integrity during physical machining and to cross-validate micro-scale simulation predictions with empirical responses, experimental investigations on the LUAT process were performed in this chapter.

### 3.5. Model Verification

In order to quantitatively verify the accuracy and reliability of the established cutting model, the pulsed laser–ultrasonic vibration-assisted cylindrical turning experiment of SiCp/Al composites was carried out. The long half-axis of the ultrasonic elliptical vibration trajectory moves along the direction of cutting depth. The dynamic constitutive parameters of 6061 Al matrix used in finite element simulation are shown in Table 4.

**Figure 7:**
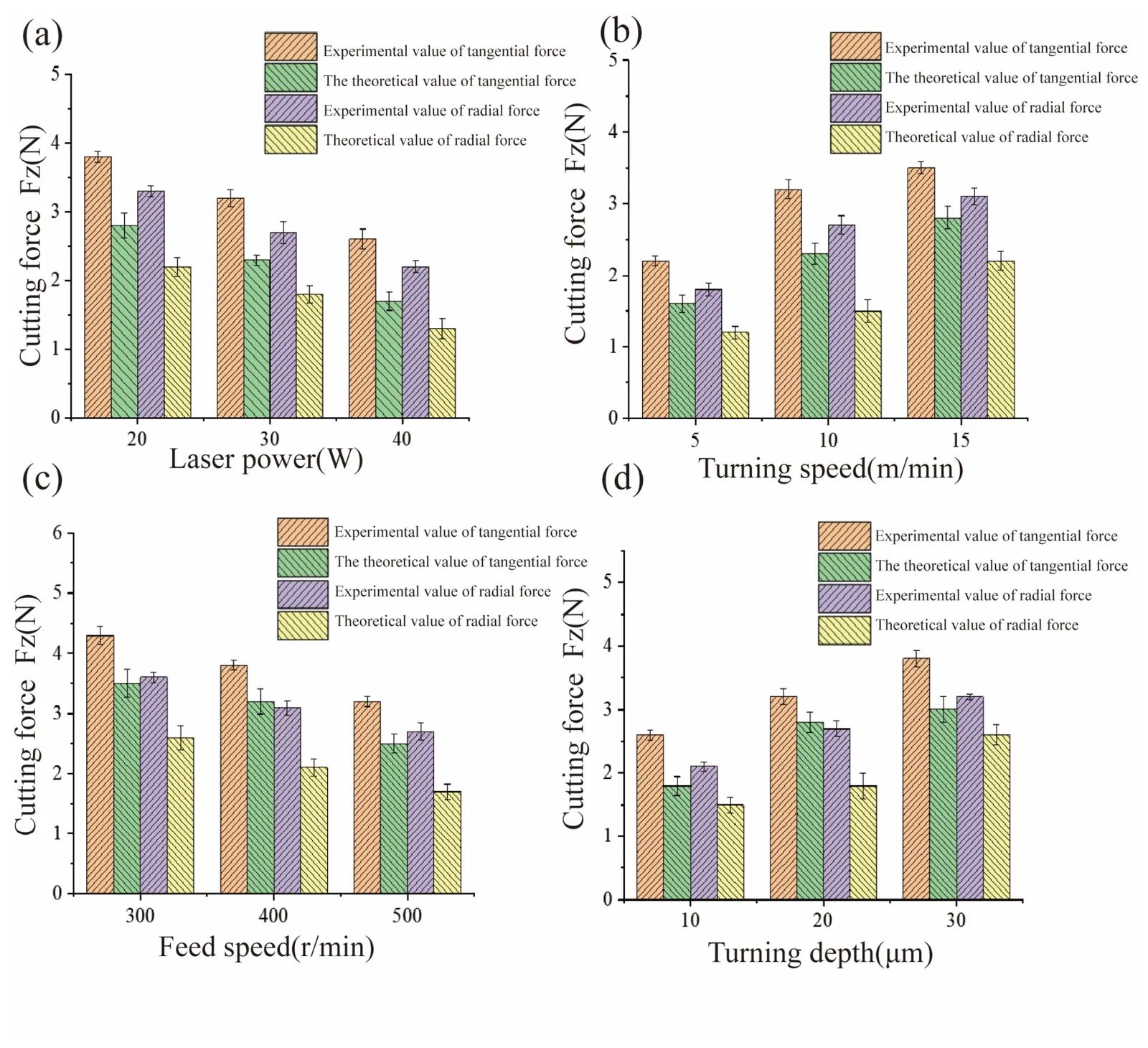
Comparison between theoretical and experimental values of cutting forces under different machining parameters: (**a**) laser power; (**b**) turning speed; (**c**) feed speed; (**d**) turning depth.

Figure 7 shows the quantitative comparison between the experimental cutting force and the theoretical simulation value under pulsed laser–ultrasonic vibration-assisted cutting. It can be seen from the figure that as the laser power increases from 20 W to 40 W, the tangential force and radial force gradually decrease. As the spindle speed increases from 300 r/min to 500 r/min, the tangential force and radial force gradually decrease. When the cutting depth increases from 10 μm to 30 μm and the feed rate increases from 5 mm/min to 15 mm/min, the tangential force and radial force increase with the increase in feed rate.

The variation law of the simulated prediction value and the experimental measurement value under various parameters maintains good consistency. The deviation in the simulated value, which is slightly lower than the experimental value, is mainly attributed to the following mechanisms: (1) In the actual machining, the blunt edge passivation of the tool tip, the micro-vibration caused by the weak stiffness of the machine tool structure and the local micro-adhesion phenomenon of the tool–chip contact interface increase the measured resistance. (2) The geometric morphology of SiC particles in the actual microstructure presents sharp and irregular polygons. Compared with the circular/polygonal homogeneous assumption simplified by the theoretical model, the local stress concentration around the sharp corners of the real polygon and the pushing resistance of the cutting tool are significantly higher. (3) The energy absorption distribution of the micro-Gaussian spot of the pulsed laser has transient locality, and there is a slight difference between the actual material heat-affected layer and the theoretical macroscopic isothermal assumption.

## 4. Results and Discussion

The material removal mode in cutting processing is determined by the adopted specific energy-field-assisted process and directly determines the dynamic cutting load and surface integrity. To investigate the influence rule of different processing methods on cutting force, this study carried out experiments on PLAT and LUAT based on the experimental parameters listed in Table 5. The evolution trend of machined surface roughness under different process parameters was explored, and the microscopic damage morphology was observed. Through a comprehensive comparison of stress state, surface roughness and damage forms under multi-energy-field assistance, the surface damage mechanism of composite materials under the action of composite energy fields is clarified.

### 4.1. Experimental Settings

**Figure 8:**
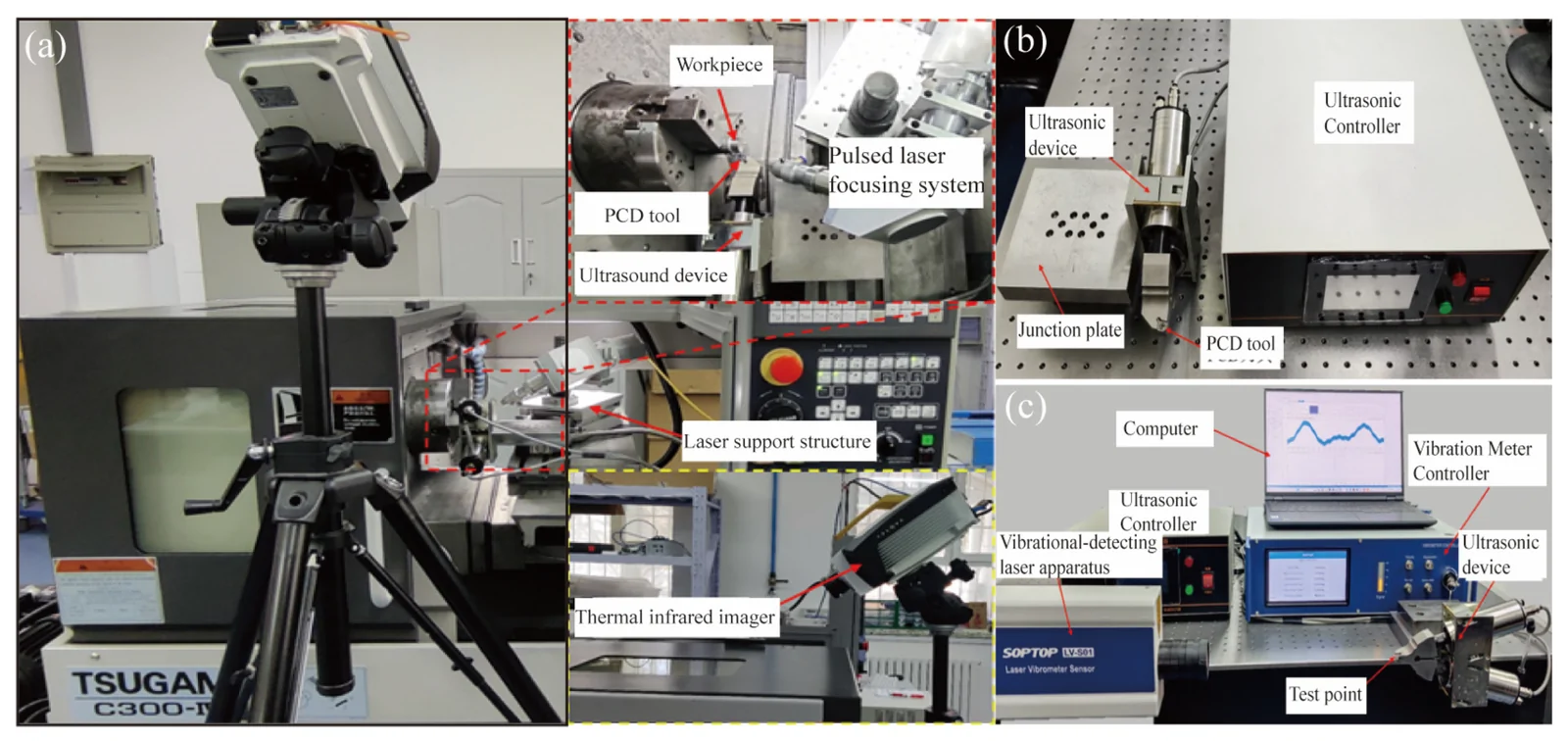
Experimental setup for pulsed laser-ultrasonic vibration composite assisted cutting: (**a**) the overall machining system and online cutting temperature monitoring device (including the CNC machine tool, pulsed laser focusing system, ultrasound device, and thermal infrared imager); (**b**) the ultrasonic vibration generation and control system; (**c**) the ultrasonic amplitude measurement and calibration system based on a laser vibrometer.

Figure 8 displays the experimental setup for pulsed laser–ultrasonic vibration hybrid cutting, constructed around a TSUGAMI C300-IV (HK, China) precision CNC automatic lathe, a 2D elliptical ultrasonic vibration module, and a pulsed laser source. The fiber laser source delivered nanosecond pulses at a 1064 nm wavelength, 240 ns pulse duration, and 1000 kHz repetition rate, featuring a maximum power of 110 W (adjustable from 10% to 100%) and a focused spot size up to 80 μm. Both the pulsed laser unit and an ultrasonic transducer—operating at 39.8 kHz with amplitudes of 2 μm (*X*-axis) and 5 μm (*Z*-axis)—were mounted on the *Z*-axis guide rail. To validate simulated force predictions, dynamic cutting forces were recorded in real time using a Kistler 9109AA 3D (Winterthur, CH, Switzerland) piezoelectric dynamometer. Transient thermal-field evolution within the cutting zone was monitored via non-contact infrared thermography using a TELOPS FAST M200 (Chengdu, China) imager. Surface topographies were mapped using a Zygo NewView 9000 (Middlefield, CT, USA) white light interferometer, taking the arithmetic mean height (Sa) as the primary metric for 3D surface evaluation. To minimize macroscopic sampling errors and reliably capture parameter-dependent roughness trends, an axial equidistant sampling protocol was implemented. Three distinct points were evaluated along the specimen length for each parameter set, and their average Sa value served as the final roughness measurement. Microscopic surface damage produced by CT, PLAT, and LUAT was analyzed via high-resolution scanning electron microscopy (JEOL JSM-IT500 SEM, Hefei, China). To test the amplitude of the two-dimensional elliptical ultrasonic vibration device, a Doppler laser vibrometer (LV-S01, Sunnyinnovation Optical Intelligence, zhejiang, China) was used to measure the axial amplitude and the lateral amplitude.

### 4.2. Influence of Process Parameters on Cutting Force

To further clarify the parameter evolution characteristics under the composite mechanism of special energy fields, the force variation laws of PLAT and LUAT under different process parameters were compared. It should be noted that during the machining of high-volume-fraction SiCp/Al composites by CT, the tool is subjected to severe mechanical impact, leading to tool breakage and abnormal wear, which makes it difficult to stably obtain continuous cutting data over a wide parameter range [38]. Meanwhile, to focus on exploring the synergistic removal efficiency enhancement mechanism of the laser–vibration dual-energy field, the subsequent parameter discussion is mainly concentrated on the comparison between PLAT and LUAT.

**Figure 9:**
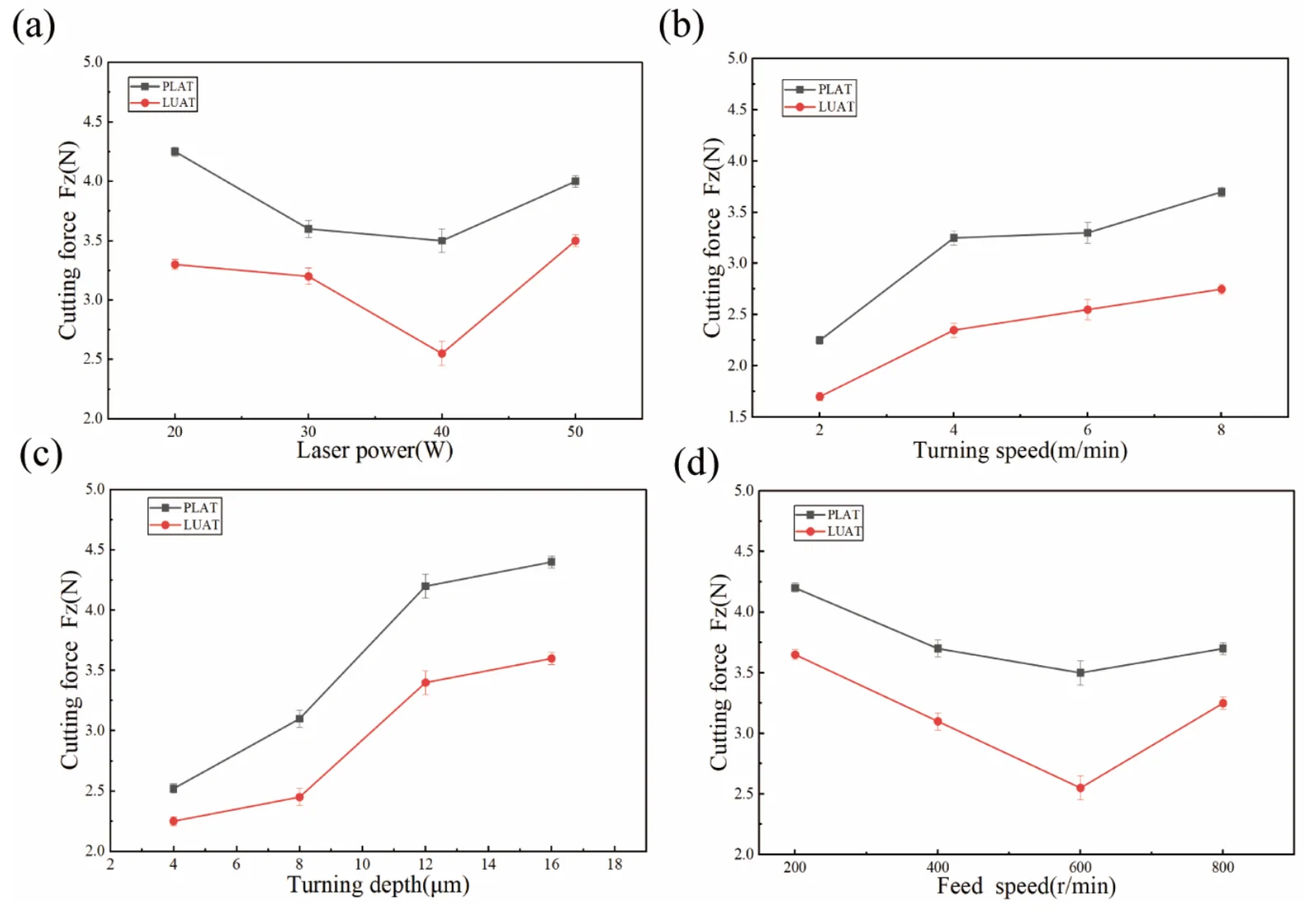
Variation in cutting force under different process parameters. (**a**) laser power, (**b**) turning speed, (**c**) turning depth, and (**d**) feed speed.

Figure 9a illustrates the variation pattern of cutting force under different laser powers. As laser power increases, the cutting forces of both PLAT and LUAT processes exhibit an evolutionary trend of decreasing first and then increasing, reaching a minimum value when the laser power is 40 W. This macroscopic mechanical behavior reflects the competitive mechanism between matrix thermal softening and tool–chip interface deterioration in the cutting zone: when the laser power is lower than 40 W, the input thermal energy field dominates the thermal softening of the aluminum matrix, significantly reducing the yield strength and deformation resistance of the material, which leads to a substantial decrease in cutting force as power increases. This finding is consistent with the analysis of the effect of laser heat source on the temperature field of the cutting zone and material softening in the preceding finite element simulation. However, when the power exceeds 40 W, excessive heat input leads to an overly high temperature in the cutting zone. This not only causes local overheating and viscous flow of the aluminum matrix and intensifies adhesion and friction at the tool–chip interface but also accelerates the thermo-mechanical wear of the tool, resulting in a counterintuitive rebound of cutting force. In addition, across the full power range, the cutting force under the LUAT mode is always significantly lower than that of PLAT, which benefits from the high-frequency intermittent cutting characteristic introduced by the ultrasonic kinetic energy field. The high-frequency intermittency weakens the average cutting force. Compared with PLAT, the average cutting force reduction achieved by LUAT during the dynamic evolution of laser power reaches 14%.

Figure 9b presents the influence pattern of spindle speed on cutting force. As the spindle speed increases, the cutting force shows a trend of decreasing first and then increasing, with the inflection point located at 600 r/min. When the speed is lower than 600 r/min, the increase in speed intensifies the heat generation from plastic shear deformation and the accumulation of cutting heat, where thermal softening dominates and suppresses the tool adhesion phenomenon at low speeds, resulting in a decrease in cutting force; when the speed exceeds 600 r/min, the material removal volume per unit time increases significantly, which aggravates mechanical impact loads. Meanwhile, the fracture and spalling of SiC particles intensify at high speeds, and hard free particles cause severe ploughing and abrasive wear on the tool surface, leading to a re-increase in cutting force. Notably, when the spindle speed increases from 600 r/min to 800 r/min, the difference in cutting force values between PLAT and LUAT shows an inward convergence trend. This is because the increase in cutting linear velocity weakens the high-frequency periodic separation effect of ultrasonic high-frequency vibration, but LUAT still exhibits an average cutting force reduction advantage of 12% over the entire speed range.

Figure 9c illustrates the evolution of dynamic cutting force with cutting depth. The cutting forces under both PLAT and LUAT exhibit a monotonic increase with cutting depth, with the LUAT curve consistently remaining lower than that of PLAT. Notably, when the cutting depth increases to 12 μm, the cutting forces in both processes display a pronounced step-like increase. This experimental trend aligns well with the spatial gradient characteristics of the transient cutting temperature field predicted by the finite element simulation in Section 3.2 (Figure 5). Numerical analysis indicates that pulsed laser preheating induces a localized thermal softening depth on the workpiece surface. When the cutting depth reaches 12 μm, the tool tip likely penetrates beyond the primary thermally softened boundary into the deeper, less-softened composite zone with higher matrix flow stress, thereby elevating cutting resistance. Concurrently, in heterogeneous ${\mathrm{SiC}}_{p}/\mathrm{Al}$ composites, such abrupt load variations may also be influenced by microstructural factors. At higher cutting depths, the enlarged tool–workpiece contact area substantially increases the probability of stochastic tool–particle collisions, cutting edge micro-chipping, and mechanical entrapment of fractured debris along the tool flank. Consequently, while the sudden force escalation at 12 μm is consistent with the simulated thermal softening boundary, it reflects the coupled manifestation of thermal gradient transitions and stochastic multiphase cutting interactions.

Figure 9d demonstrates the variation pattern of cutting force under different feed rates. As the feed rate increases, the cutting forces of both PLAT and LUAT processes show a significant upward trend. The core physical mechanism of this phenomenon lies in the following: on the one hand, the increase in feed rate directly increases the cross-sectional area of the cutting layer and the material removal volume per unit time, which intensifies the macroscopic mechanical friction and shear resistance at the tool–workpiece interface; on the other hand, the increase in feed rate shortens the irradiation residence time of the pulsed laser spot on the unit area of the workpiece surface to be processed. This is consistent with the modulation effect of heat source moving speed on thermal energy-field accumulation in the previous finite element temperature-field simulation; that is, the shortened energy input time reduces the transient peak temperature in the cutting zone and thins the depth of the thermal softened layer, significantly weakening the thermal softening effect at the interface between the aluminum matrix and SiC particles. Consequently, SiCp/Al composites exhibit more obvious cold–hard removal characteristics, which jointly push up the macroscopic cutting load. Notably, under the condition that the thermal softening effect attenuates with the increase in feed rate, the LUAT mode still achieves an average cutting force control advantage of 16% compared with PLAT. This indicates that the high-frequency periodic interrupted cutting and interface friction reduction effect of the ultrasonic kinetic energy field can effectively compensate for the increase in cutting resistance caused by insufficient thermal softening, which further confirms the advantage of laser–vibration dual-energy-field-assisted composite cutting in improving processability.

**Figure 10:**
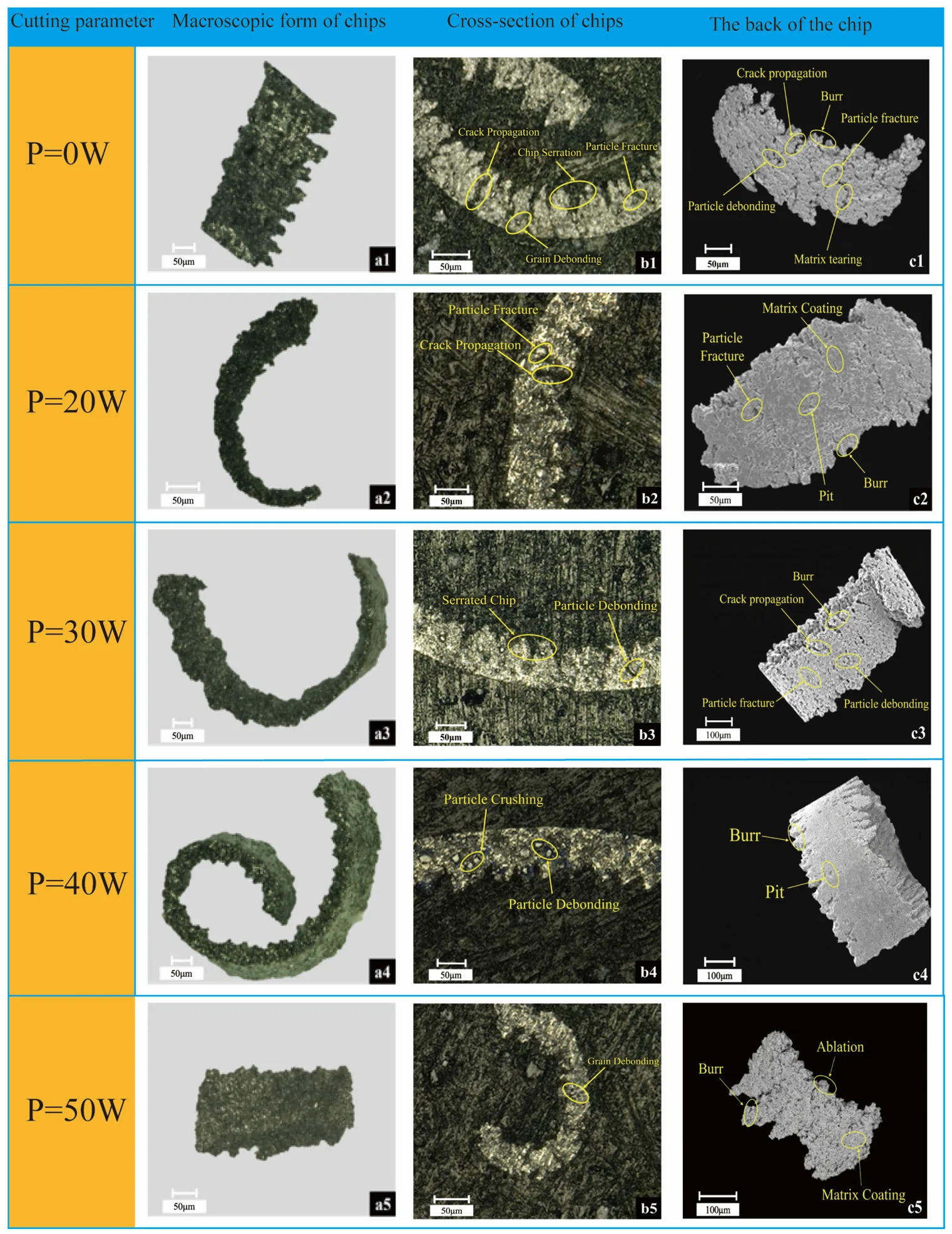
Macroscopic and microscopic morphologies of chips under different laser powers (P = 0–50 W): (**a1**–**a5**) macroscopic forms; (**b1**–**b5**) cross-sections; (**c1**–**c5**) back surfaces of the chips.

The evolution of chip morphology directly reflects the stress state and cutting force variations within the cutting deformation zone. Figure 10 illustrates the evolution of chip morphology under different laser powers obtained through single-factor experiments. At a low pulsed laser power (0 W), crack propagation within the chip is deep, and the serration is severe. The frequent brittle fracture of particles leads to deformation instability in the primary shear zone, keeping the cutting force at a high level, accompanied by significant dynamic fluctuations. As the laser power gradually increases to 40 W, the thermal softening effect of the Al matrix significantly enhances the plastic fluidity of the material. The deformation of the shear slip band tends to be uniform, chip serration is weakened, and curling becomes more adequate. Consequently, the shortened tool–chip contact length directly leads to a substantial reduction in both the principal cutting force and the feed resistance. However, when the laser power is further increased to above 50 W, excessive heat input triggers severe chip ablation and matrix adhesion. This increases the tool–chip friction coefficient, limits further reduction in the cutting force, and results in a rebound of the cutting force.

### 4.3. Evolution Law of Surface Roughness of Machined Surface

The three-dimensional morphology and arithmetical mean height (Sa) of the machined surface are the ultimate macro-geometric and physical indicators that map the material removal mode of SiCp/Al composites and the regulation effect of the multi-energy-field synergistic mechanism. To elucidate the shape control mechanism of energy-field intervention on surface defects, validate the micro-generation prediction of the finite element analysis (FEA) surface morphology presented in Section 3.3 at the macro-experimental level, determine the cutting process parameters with optimal surface integrity, and analyze the nonlinear modulation law of process parameters on surface quality, this section evaluates the surface quality obtained under different process parameters through systematic experiments.

**Figure 11:**
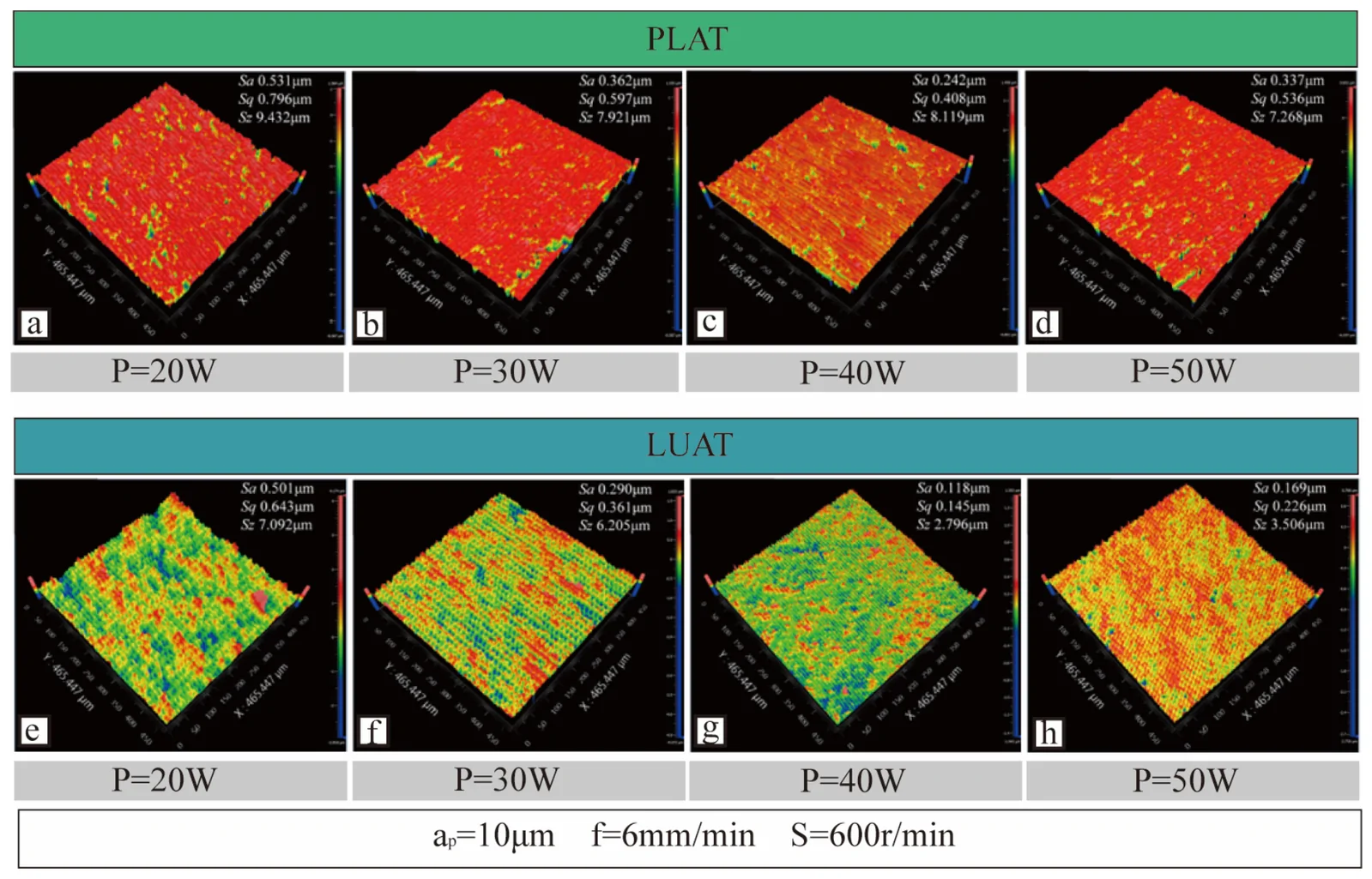
Effects of different laser powers on 3D surface topographies machined by PLAT and LUAT under fixed cutting parameters ($a_{p}$ = 10 μm, f = 6 mm/min, S = 600 r/min): (**a**–**d**) topographies of PLAT at P = 20 W, 30 W, 40 W, and 50 W; (**e**–**h**) topographies of LUAT at P = 20 W, 30 W, 40 W, and 50 W.

Figure 11 intuitively compares the three-dimensional surface morphologies of PLAT and LUAT specimens fabricated under different laser powers. In PLAT mode, the workpiece surface suffers severe damage, with densely distributed spalling pits left by particle detachment and deep plowing grooves. In contrast, the machined surface obtained via LUAT mode exhibits a unique microscopic vibration mark texture characteristic of high-frequency ultrasonic impact, and both the depth and area of residual spalling pits are significantly reduced. With the increase in laser power, the surface defects generated by the two processes follow an evolution pattern of decreasing first and then increasing, and the smoothest surface is obtained when the laser power is 40 W.

The aforementioned macroscopic evolution of surface morphology confirms the simulation prediction of the material microscopic removal mechanism obtained from the finite element simulation presented earlier. Its physical essence lies in the co-regulation of microscopic removal behavior by thermal softening flow and interface adhesion state under multi-energy-field coupling: when the laser power is below the critical value of 40 W, the increase in energy input raises the temperature of the cutting zone, leading to the sufficient thermal softening of the aluminum matrix. This conclusion is highly consistent with the microscopic removal simulation results in Section 3.2. The aluminum matrix with high plastic flow has a good coating and load slow-release effect on the hard SiC particles under tool extrusion, which effectively inhibits the stress concentration and the large-scale peeling and debonding of the particle interface, thus greatly reducing the secondary plough scratches caused by the falling off of the hard particles. However, when the laser power exceeds 40 W, although the deformation resistance of the material is further reduced, the excessive cutting heat causes the aluminum matrix to overheat and adhere, causing serious tool–chip/tool–work interface bonding. The secondary scratching and tearing of the machined surface is caused by adhesion, and the high temperature accelerates the thermo-mechanical wear and passivation of the tool edge, resulting in the increase in surface damage and defect rebound.

**Figure 12:**
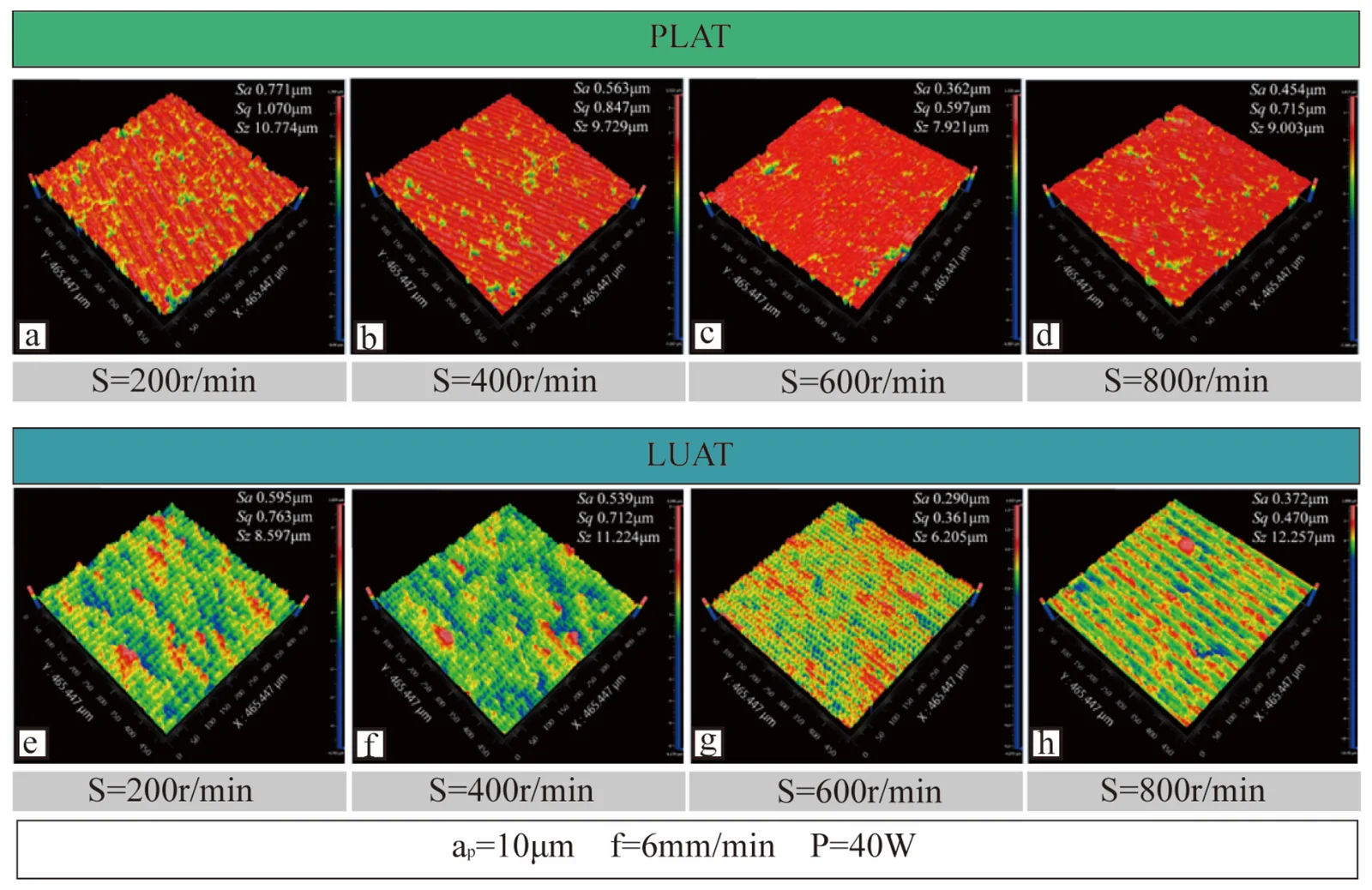
Effects of different spindle speeds on 3D surface topographies machined by PLAT and LUAT under fixed parameters ($a_{p}$ = 10 μm, *f* = 6 mm/min, *P* = 40 W): (**a**–**d**) topographies of PLAT at *S* = 200, 400, 600, and 800 r/min; (**e**–**h**) topographies of LUAT at *S* = 200, 400, 600, and 800 r/min.

Figure 12 illustrates the evolution law of surface roughness of workpieces processed by PLAT and LUAT under different spindle speeds. When the spindle speed is lower than 200 r/min, the surface damage induced by both processing technologies is relatively severe, with deep residual spalling pits and generally high surface roughness values. This is mainly attributed to the low shear strain rate during material removal at low cutting speeds. The stress release of SiC particles under tool extrusion proceeds slowly, which fails to effectively mitigate the particle–matrix interface stress concentration revealed in the preceding finite element simulation, and further aggravates the interface debonding of SiC particles and macroscopic plowing behavior. Meanwhile, the Al matrix undergoes severe plastic tearing at low speeds, which significantly degrades surface micro-quality.

When the spindle speed is increased to 600 r/min, the surface roughness under PLAT and LUAT conditions is reduced to 0.362 μm and 0.290 μm, respectively. This velocity range coordinates the material removal rate and the energy-field input effect and reduces the probability of crack initiation and propagation at the particle interface. Therefore, most of the SiC particles are successfully discharged or discharged in time in the form of high-strain-rate local micro-fracture predicted by finite element simulation, which greatly reduces the negative impact of particle spalling pits on surface integrity.

However, as the spindle speed continues to increase, the surface roughness obtained by both processing technologies rises again. This phenomenon is constrained by the dual effects of aggravated tool wear at high spindle speeds and degradation of the ultrasonic high-frequency separation effect: on the one hand, high spindle speed significantly increases the friction work per unit time and the volume of removed material, leading to aggravated thermo-mechanical wear of the tool. The blunted tool edge causes strong extrusion and micro-tearing on the workpiece surface; on the other hand, excessive cutting linear speed weakens the advantage of the high-frequency periodic intermittent cutting of LUAT, which is mutually verified by the convergence phenomenon of the cutting force difference between PLAT and LUAT measured in the previous section, and degrades the cutting state to conventional continuous cutting, consequently leading to the re-deterioration of processed surface quality.

**Figure 13:**
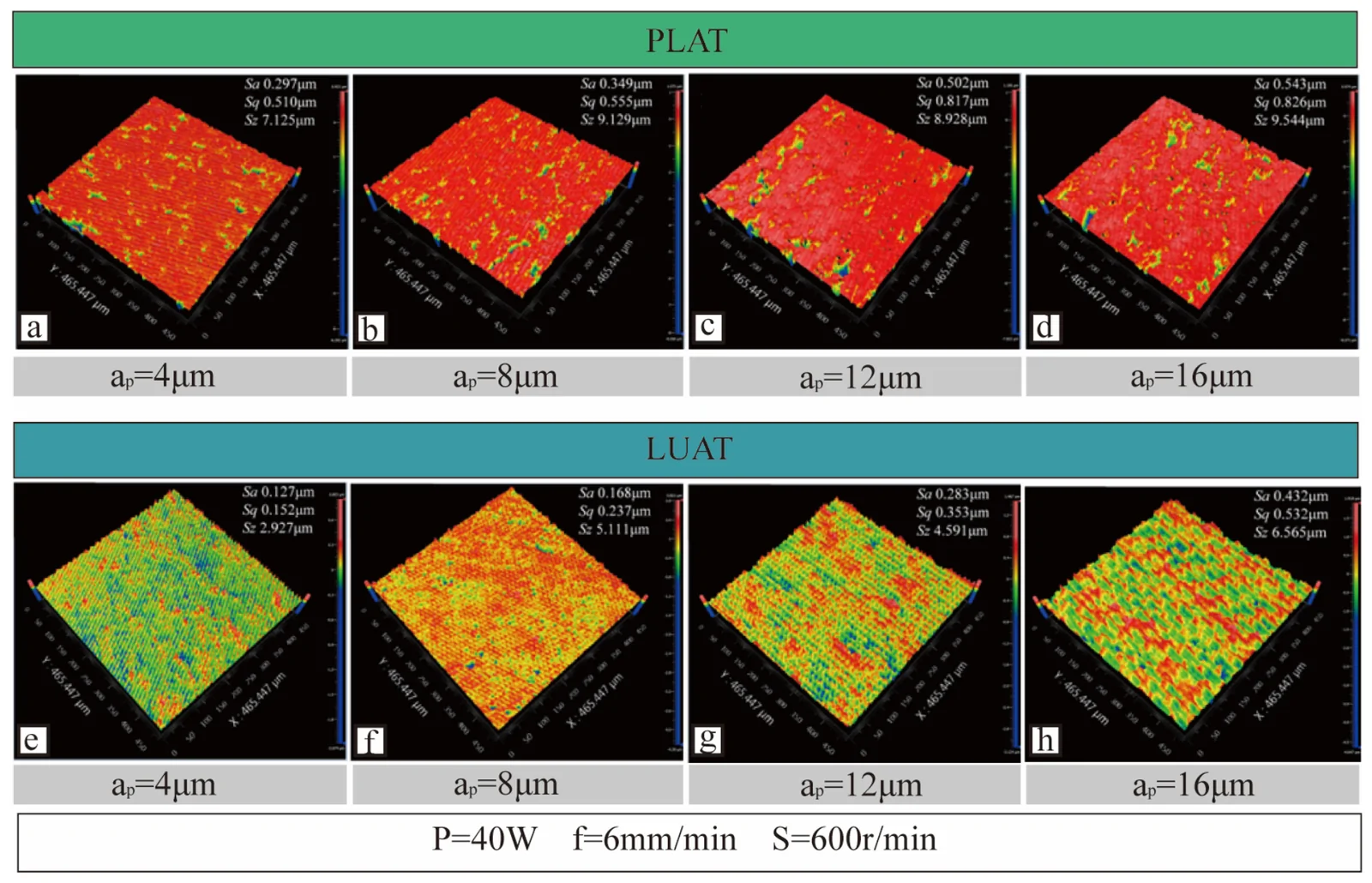
Effects of different turning depths on 3D surface topographies machined by PLAT and LUAT under fixed parameters (*P* = 40 W, *f* = 6 mm/min, *S* = 600 r/min): (**a**–**d**) topographies of PLAT at $a_{p}$ = 4, 8, 12, and 16 μm; (**e**–**h**) topographies of LUAT at $a_{p}$ = 4, 8, 12, and 16 μm.

Figure 13 shows the evolution of surface roughness of PLAT and LUAT workpieces under different cutting depths. When the cutting depth is 4 μm, the surface roughness under both processing conditions remains at a low level. Compared with PLAT, LUAT showed a significant reduction in peeling pits and a significant increase in surface roughness. This is mainly attributed to the high-frequency intermittent cutting mechanism of the ultrasonic kinetic energy field: its pulse impact promotes the high-strain-rate local micro-fracture of the hard SiC particles predicted by the finite element simulation, and the broken particles are discharged in time with the chips, thus effectively inhibiting the large-scale overall debonding of the particles and the subsequent macroscopic ploughing behavior.

However, as the cutting depth continues to increase, surface damages and defects under both processing methods exhibit an increasing trend, and the surface roughness rebounds and rises accordingly. This deterioration trend of macroscopic surface quality physically confirms the simulation conclusion regarding the spatial gradient of transient temperature field in the finite element simulation in Section 3.2 and mutually corroborates the observed cutting force jump measured in Figure 9 cof the previous section. The thermal energy field applied by pulsed laser has a remarkable characteristic of high-temperature softening in the surface layer and gradient attenuation towards the inner layer. As the cutting depth increases, the cutting zone gradually extends into the region inside the workpiece with insufficient energy conduction, and the thermal softening effect of the substrate attenuates accordingly. In this cold–hard removal state, the aluminum matrix loses its rheological coating on SiC particles and load buffering effect, and the hard particles undergo severe macroscopic debonding and pull-out under the intense mechanical extrusion of the cutting tool; the detached hard particles cause significant secondary ploughing damage between the cutting tool and the workpiece, which in turn leads to a significant increase in the processed surface roughness.

**Figure 14:**
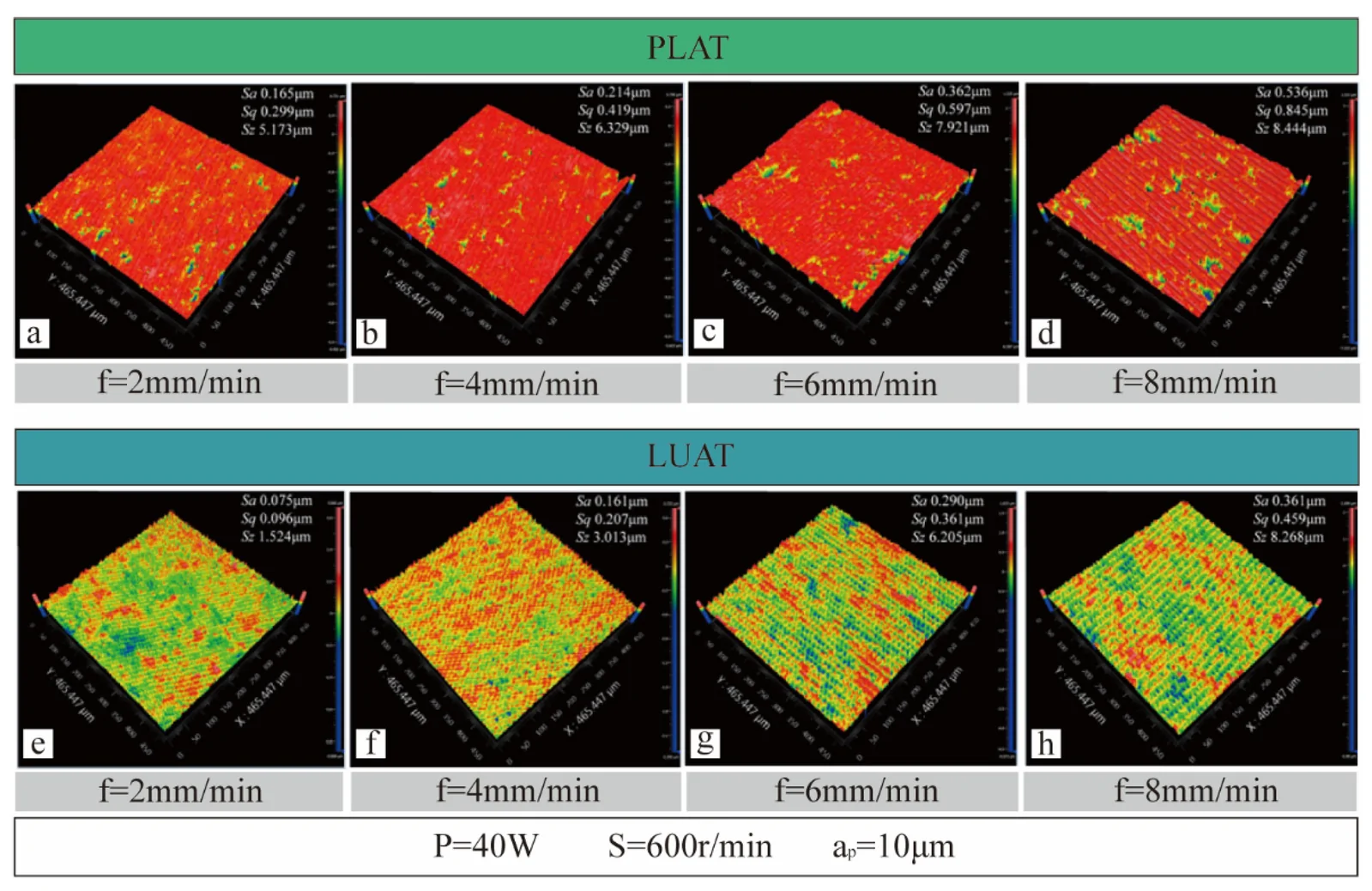
Effects of different feed rates on 3D surface topographies machined by PLAT and LUAT under fixed parameters ($a_{p}$ = 10 μm, S = 600 r/min, P = 40 W): (**a**–**d**) topographies of PLAT at f = 2, 4, 6, and 8 mm/min; (**e**–**h**) topographies of LUAT at f = 2, 4, 6, and 8 mm/min.

Figure 14 illustrates the evolution of machined surface roughness under PLAT and LUAT across different feed rates. At a feed rate of 2 mm/min, both cutting modes achieve their lowest surface roughness values among the tested levels, recording $S_{a}$ of 0.165 μm for PLAT and 0.075 μm for LUAT—a substantial reduction of 54.5% achieved by the hybrid process. Across the tested parametric range, feed rate exerts a dominant influence on surface roughness, with lower feed rates yielding markedly improved surface finish.

This variation in surface roughness corresponds to the heat accumulation dynamics of the moving laser beam modeled in Section 3.2, corroborating the cutting force trends shown in Figure 9d. At lower feed rates, the elevated pulse overlap ratio and prolonged laser residence time facilitate sufficient thermal accumulation within the cutting zone. The resulting matrix softening and enhanced thermoplastic rheology help accommodate hard SiC particles, mitigating severe interfacial stress concentration and stabilizing material removal. Conversely, higher feed rates shorten the laser interaction duration and increase the material removal volume per unit time, weakening the thermal softening effect. Under these less-softened cutting conditions, limited matrix compliance favors particle fracture and debonding. Dislodged hard debris becomes trapped at the tool–workpiece interface, exacerbating secondary plowing scratches and spalling pits, which markedly elevates the resulting surface roughness.

**Figure 15:**
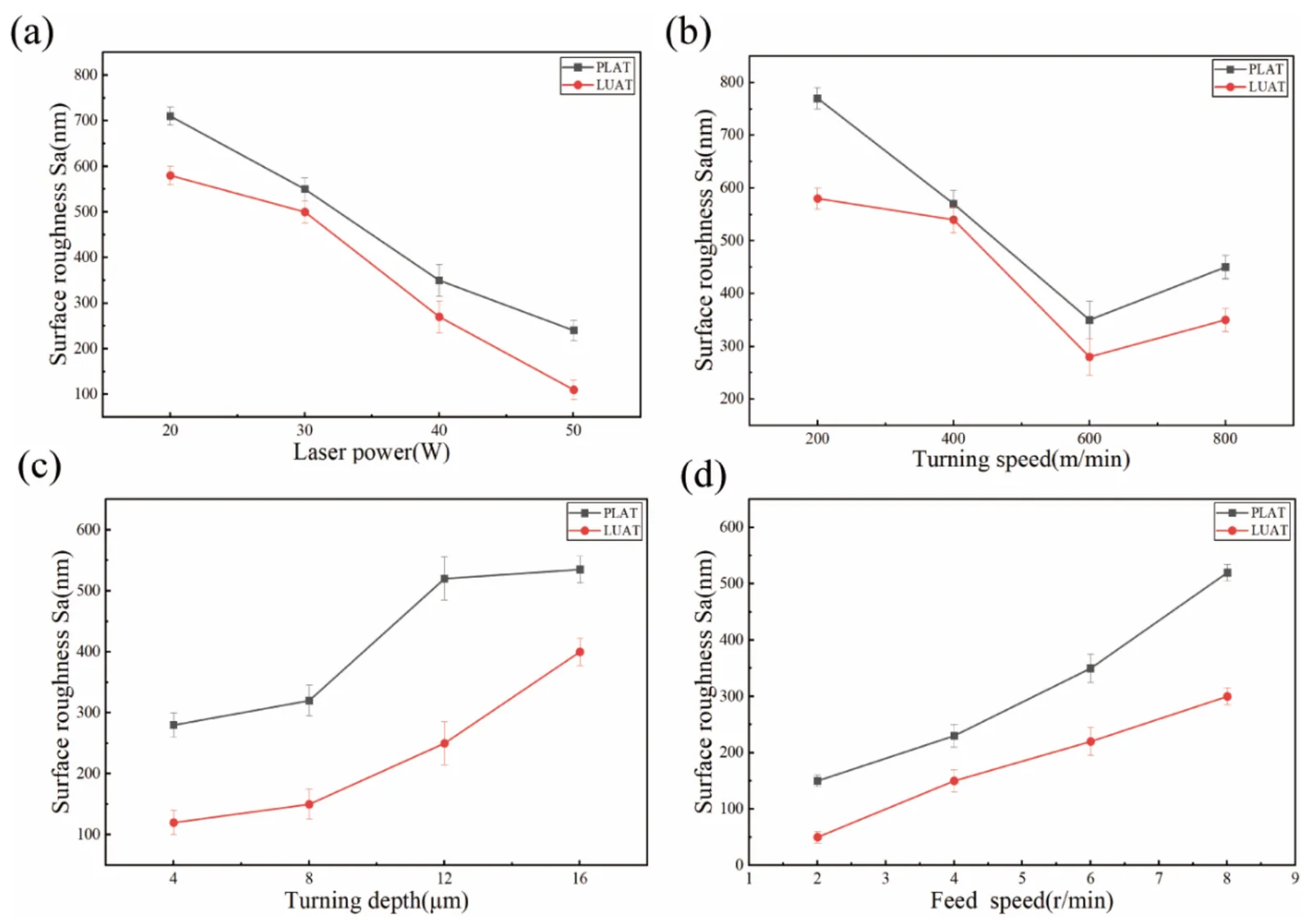
Effects of machining parameters on surface roughness ($S_{a}$) under PLAT and LUAT conditions: (**a**) laser power, (**b**) turning speed, (**c**) turning depth, and (**d**) feed speed.

In conclusion, LUAT produces superior surface topographies compared with alternative methods, as evidenced in Figure 15. Under the optimized settings of 2 mm/min feed rate, 600 r/min spindle speed, 4 μm depth of cut, and 40 W laser excitation, surface roughness reached a minimum of approximately 0.075 μm, representing a 56% improvement over PLAT.

### 4.4. Micro-Scale Surface Damage Mechanism

**Figure 16:**
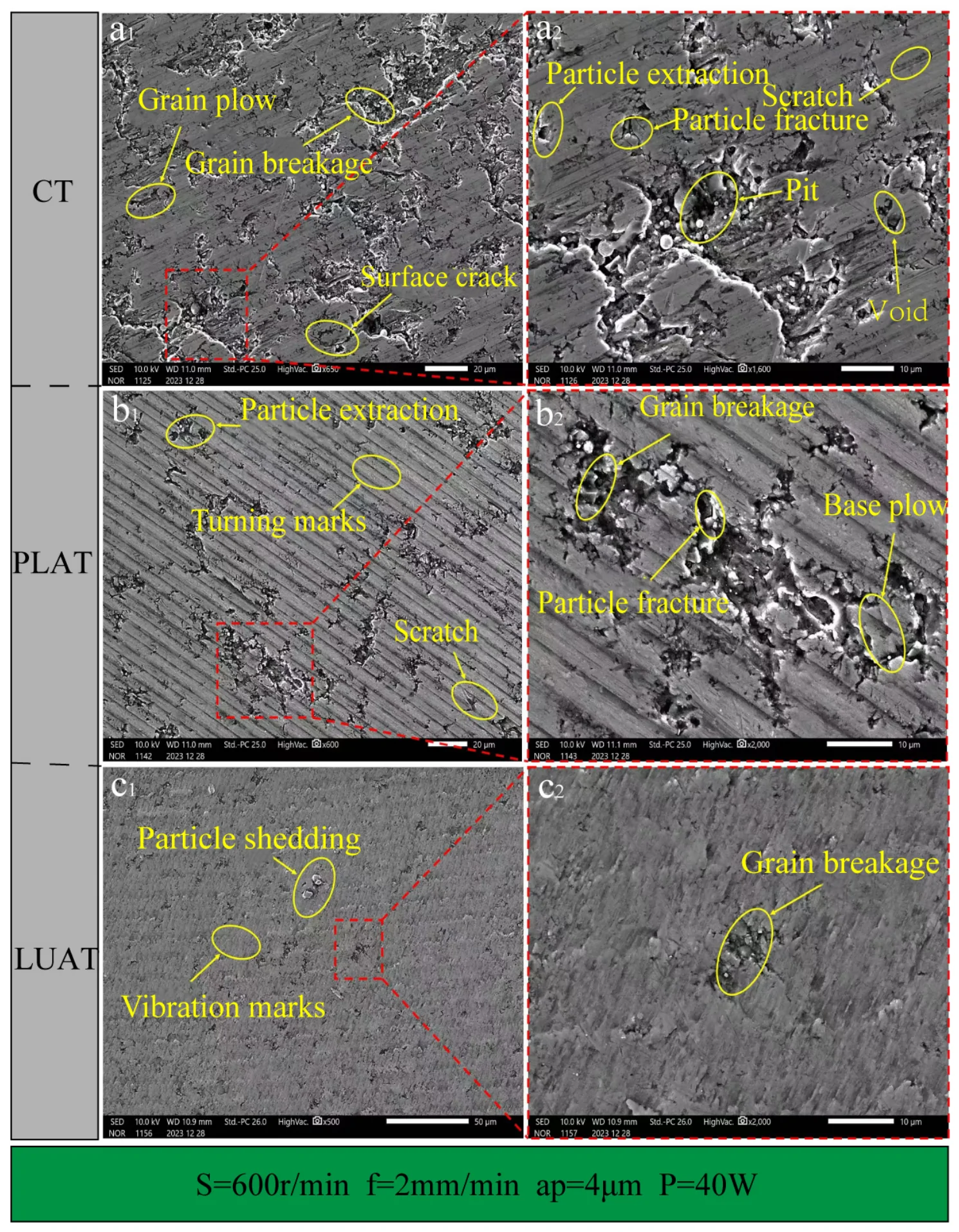
SEM images of machined surface morphologies under different machining conditions (S = 600 r/min, f = 2 mm/min, $a_{p}$ = 4 μm, P = 40 W): (**a1**,**a2**) CT; (**b1**,**b2**) PLAT; (**c1**,**c2**) LUAT.

Figure 16 compares the machined surface damage morphologies of ${\mathrm{SiC}}_{p}/\mathrm{Al}$ composites across the three cutting modes. Under conventional turning (CT), the surface is severely degraded by deep spalling pits, intra-particle microcracks, and prominent plowing grooves along the cutting path (Figure 16(a1)). The high-magnification view in Figure 16(a2) reveals fractured SiC fragments and interfacial debonding voids left by completely torn-out particles. Here, unaccommodated stress concentration leads to macroscopic matrix tearing and severe secondary plowing from trapped abrasive debris.

Under pulsed laser-assisted turning (PLAT), localized thermal softening reduces the shear strength of the Al matrix, transforming the brittle failure mode into continuous plastic flow and ductile plowing marks (Figure 16(b1)). The compliant matrix cushions the hard particles, mitigating large-scale spalling into shallow microscopic defects (Figure 16(b2)). However, continuous tool–workpiece contact under thermal softening still leaves pronounced ductile plowing ridges.

In contrast, hybrid laser–ultrasonic turning (LUAT) yields superior surface integrity, characterized by uniform, high-frequency micro-textures rather than macroscopic tearing (Figure 16(c1)). As evidenced in Figure 16(c2), this improvement stems directly from the thermo-mechanical synergy of the hybrid fields. High-frequency ultrasonic mechanical pulses concentrate stress locally within the hard SiC particles, inducing controlled microcracking instead of catastrophic interfacial debonding. Concurrently, the laser-softened Al matrix exhibits enhanced thermoplastic rheology under high-frequency tool hammer compaction, which embeds the fractured SiC fragments firmly beneath the surface. This coupled mechanism eliminates macroscopic voids and whole-particle pull-out, while periodic tool–workpiece separation effectively suppresses abrasive debris entrapment and secondary plowing scratches.

**Figure 17:**
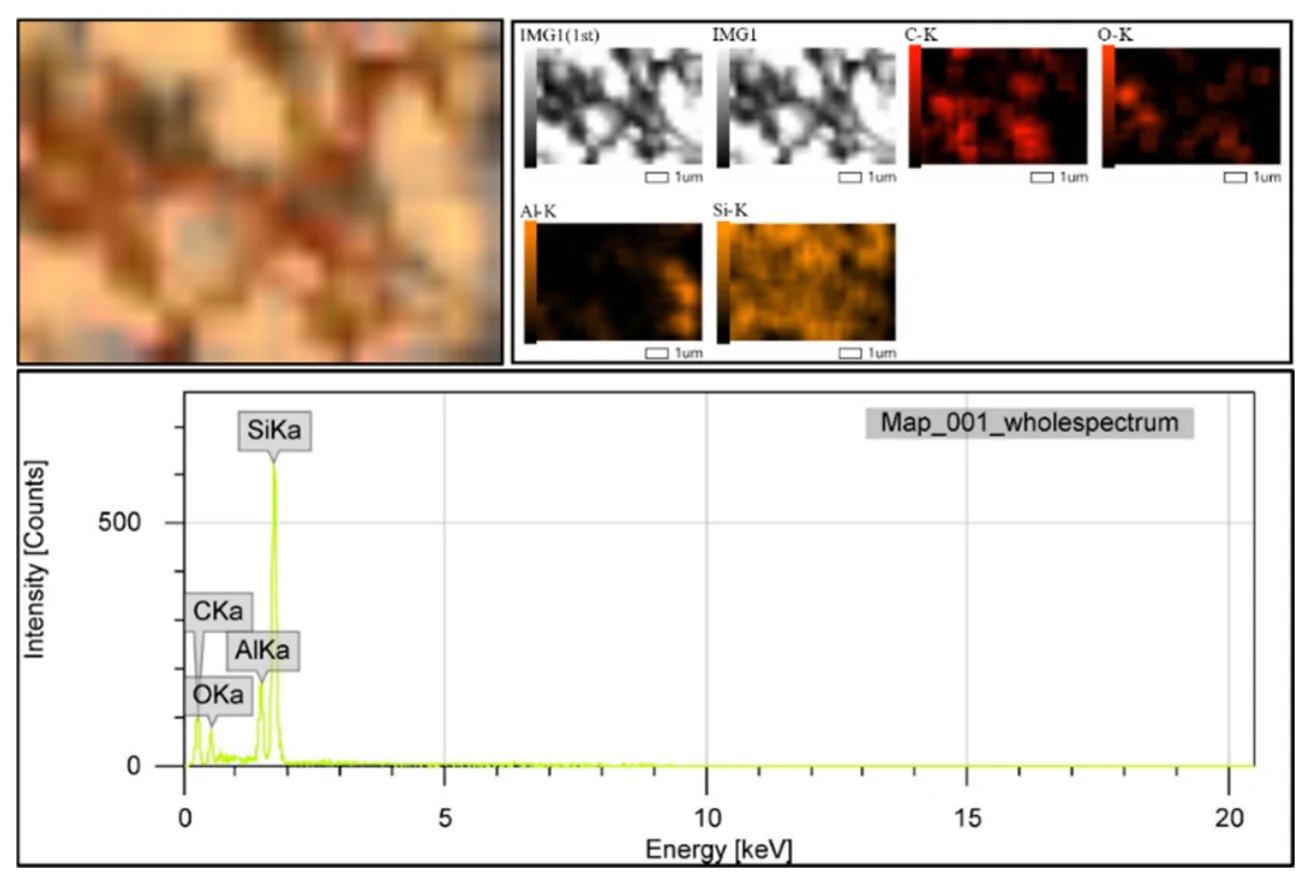
Energy-dispersive spectroscopy analysis of elements inside the pit.

To characterize the superficial phase distribution under LUAT, energy-dispersive X-ray spectroscopy (EDS) mapping was performed across the representative surface regions, as depicted in Figure 17. The elemental maps reveal localized Si signals corresponding to residual micro-pits, while the surrounding flat regions exhibit predominant Al enrichment. These elemental distributions provide supportive experimental evidence for the transient thermo-mechanical behavior modeled in Section 3.3 Figure 6c. During LUAT, laser-induced thermal softening lowers the flow stress of the Al matrix, while high-frequency ultrasonic impact introduces periodic, localized stress concentrations at the cutting zone. Rather than inducing catastrophic interfacial debonding or deep particle pull-out, this dynamic coupling favors localized micro-fracturing of SiC particles under high strain rates. Concurrently, the cyclic tool contact encourages localized thermoplastic flow of the softened Al matrix around the fragmented phases. This mechanism tends to suppress sharp particle protrusion, thereby mitigating secondary abrasive plowing and reducing micro-groove formation.

In summary, comparing the surface damage characteristics across the three processes demonstrates the role of coupled energy fields in governing material removal: CT is dominated by continuous mechanical stress concentration, leading to brittle fracture, severe interfacial debonding, and macroscopic spalling pits. PLAT enhances matrix plasticity via laser thermal softening, transforming the removal regime toward continuous ductile plowing and reducing particle dislodgement, though ductile tool marks remain prominent. LUAT establishes a distinct thermo-mechanical synergy. The combination of thermal softening and periodic ultrasonic separation moderates interface stress transfer, encouraging localized phase fracture and facilitating matrix plastic deformation over fragmented debris. Consequently, LUAT effectively suppresses macroscopic void formation and microcracks, yielding superior surface integrity.

## 5. Conclusions

Aiming at the specialized removal dynamics of high-volume-fraction SiCp/Al composites, this work employed a joint 2D FEA simulation and experimental methodology to clarify force responses and surface damage evolution under pulsed laser–ultrasonic vibration-assisted composite turning processing. The key results and insights are outlined as follows:

(1) Pulsed laser heating induces transient thermal softening that lowers the shear yield strength of the Al matrix, while high-frequency ultrasonic vibration establishes periodic tool–workpiece separation to reduce contact friction and relieve interface stress concentration. Driven by the competition between thermal softening and high-speed thermo-mechanical tool wear, the cutting force exhibits inflection minima at 40 W laser power and 600 r/min spindle speed. Lower feed rates increase the laser pulse overlap per unit area, maximizing the thermal softening effect and minimizing average cutting forces.
(2) Under hybrid energy fields, uncontrolled large-scale brittle spalling and complete particle pull-out are effectively suppressed. Microscopic FE simulation and SEM/EDS analyses demonstrate that mechanical pulse impacts promote localized, micro-scale brittle fracture of hard SiC particles rather than deep interfacial debonding. Concurrently, the thermally softened Al matrix undergoes periodic ultrasonic compression, facilitating continuous plastic flow that tends to embed fractured particles beneath the surface and prevents macroscopic void formation.
(3) Compared with the traditional turning with brittle erosion pits and pulsed laser-assisted turning with obvious furrow characteristics, pulsed laser–vibration-assisted turning significantly reduces secondary furrow scratches and surface microcracks. Under the condition of a feed rate of 2 mm/min, a spindle speed of 600 r/min, a cutting depth of 4 μm and a laser power of 40 W, the minimum surface roughness (Sa) is 0.075 μm, which is 56% lower than that obtained by using pulsed laser-assisted turning.

## Figures and Tables

**Table 1:** Parameters of cutting tools and composite materials.

Property	Workpiece	Cutting Tool
Density	2.9 × 10^−6^ (kg/mm^3^)	4.25 × 10^−6^ (kg/mm^3^)
Elasticity modulus	147 GPa	1211 GPa
Major component	SiC 45% Al 55%	PCD
Heat conductivity	140 W/(m k)	525 W/(m K)
Thermal expansivity	12 × 10^−6^/K	1.2 × 10^−6^/K

**Table 2:** Cutting parameters for finite element simulation.

Argument	Value
Turning speed	15.7 m/min
Turning depth	100 μm
*Z*-axis amplitude	5 μm
*X*-axis amplitude	2 μm
Angle of cutting edge	0°
Tool clearance	7°

**Table 3:** Material characteristics of 6005Al.

Parameter	Value
Density (ton/mm^3^)	2.7 × 10^−9^
Young modulus (MPa)	70,000
Poisson ratio	0.33
T_m_ (℃)	600
T_r_ (℃)	23
*A* (MPa)	270
*B* (MPa)	134
*n*	0.514
*C*	0.0082
*m*	0.703
*D*_1_	0.06
*D*_2_	0.497
*D*_3_	−1.551
*D*_4_	0.0286
*D*_5_	6.8

**Table 4:** Constitutive parameters of 6005Al.

Parameter	K (MPa)	B (MPa)	C	ς	$T_{m}$ (℃)	$T_{r}$ (°C)
Value	270	134	0.0082	0.514	600	23

**Table 5:** Experimental parameters.

Argument	Value
Laser power	20, 30, 40, 50 W
Turning speed	200, 400, 600, 800 r/min
Turning depth	4, 8, 12, 16 μm
Feed speed	2, 4, 6, 8 m/min

## Data Availability

Data will be made available upon request.

## Supplementary Materials

The following supporting information can be downloaded at: https://www.mdpi.com/article/10.3390/mi17101144/s1, Figure S1: A schematic diagram of the distance between the laser spot center and the tool tip; Figure S2: Gaussian distribution cross section of laser energy density; Figure S3: Temperature conduction model of pulsed laser assisted cutting.

## Abbreviations

The following abbreviations are used in this manuscript:

CT	Conventional turning
PLAT	Pulsed laser-assisted turning
LUAT	Pulsed laser–ultrasonic vibration-assisted composite turning
FEA	Finite element analysis
FEM	Finite element method
