# Supplementary material for: Study on the Machining Characteristics of SiCp/Al Under Pulsed Laser–Ultrasonic Vibration-Assisted Composite Turning
Source: Micromachines (Basel). 2026 Sep 30;17(10):1144. doi: 10.3390/mi17101144 (PMC13707789; doi:10.3390/mi17101144)
Supplement: Supplementary file 1 [file micromachines-17-01144-s001.zip › micromachines-4551754-supplementary.pdf]

Supplementary Materials. *Establishment of pulsed laser finite element model*

In the pulsed laser-assisted turning process, the relative distance between the laser spot center and the tool tip significantly affects the thermal softening effect in the cutting zone. Upon laser irradiation, heat conduction from the workpiece surface to the interior requires a certain response time. If this distance is too short, the surface heat cannot penetrate sufficiently into the subsurface, resulting in an inadequate thermal softening depth. Conversely, if the distance is too large, the absorbed heat will rapidly dissipate through thermal convection and radiation, thereby failing to achieve the desired softening effect. Since precisely measuring this dynamic micro-distance during actual experiments is challenging, the effective distance between the pulsed laser spot center and the tool tip is assumed to be l = 100μm in the finite element (FE) model, as illustrated in Fig. S1.

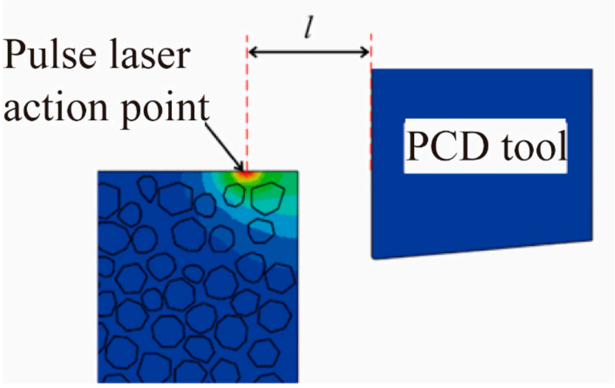

Figure S1 A schematic diagram of the distance between the laser spot center and the tool tip

The conversion of the total energy radiated by the laser on the surface of the workpiece follows the law of conservation of energy. The sum of the laser radiation energy is equal to the sum of the energy absorbed by the workpiece, the energy reflected by the workpiece and the energy transmitted through the workpiece, which can be expressed as :

$$E_0 = E_1 + E_2 + E_3 \qquad \text{(S1)}$$

In the formula, E_0 is the total energy of laser radiation ; E_1 is the total energy absorbed by the workpiece ; E_2 is the total reflected energy on the surface of the workpiece ; E_3 is the total transmission energy of the workpiece.

The total incident laser energy on the workpiece surface is primarily divided into three components: the heat absorbed and conducted into the workpiece, the energy lost to the surrounding environment through surface reflection, thermal convection, and thermal radiation, and the transmitted energy passing through the material. Because the SiCp/Al composite used in this study is opaque, the transmitted energy is zero. Therefore, the energy balance equation for the laser irradiation on the workpiece surface can be expressed as:

$$E_0 = E_1 + E_2 \qquad \text{(S2)}$$

By converting the formula (S1-S2), the relationship between the absorption rate and the reflectivity of the workpiece can be obtained :

$$\frac{E_1}{E_0}+\frac{E_2}{E_0}=R+A=1 \quad \text{(S3)}$$

In the formula, R is the workpiece absorption rate, assuming that the workpiece absorption rate does not change with the workpiece temperature ; A is the reflectivity of the workpiece.

During the pulsed laser irradiation of the workpiece, the spatial intensity of the laser beam follows a Gaussian distribution, where the intensity exponentially decays from the center of the spot toward the periphery, as illustrated in Fig. S2. Because the laser energy density is highly concentrated in the central region, approximately 86.5% of the total laser energy is distributed within a circular area of radius r_b Therefore, in the finite element simulation model, this radius r_b is defined as the effective spot radius of the pulsed laser irradiating the workpiece surface.

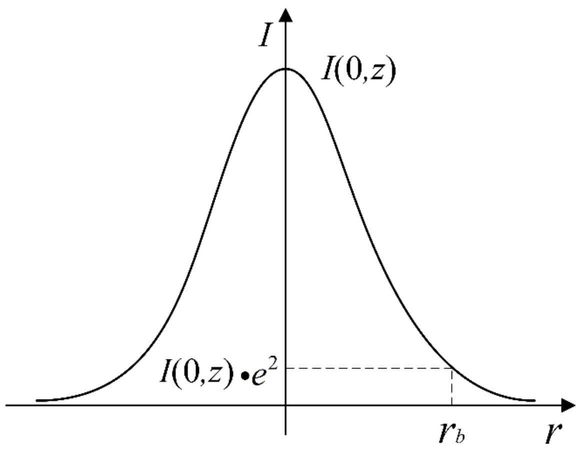

Figure S2 Gaussian distribution cross section of laser energy density

When the pulsed laser acts on the workpiece, it can be approximately considered that the laser acts on the surface area of the workpiece. Therefore, in the setting of the laser, the pulsed laser heat source is regarded as the surface heat source. The power density distribution of the pulsed laser satisfies the two-dimensional Gaussian function distribution. Therefore, when the laser is applied to the model, the power density distribution of the pulsed laser is established by Formula (S1-S4).

$$I(r,z)=I(0,z)\exp(\frac{2R^2}{r_b^2}) \quad \text{(S4)}$$

Unlike continuous laser, the heat flux density of pulsed laser is generated in a cycle, so in the formula (S1-S4), it can be expressed as follows by formula (S1-S5) :

$$I(0,z)=\begin{cases}\dfrac{P}{\pi\tau f_p r_b^2} & 0\le t\le\tau \\ 0 & \tau<t<T\end{cases} \quad \text{(S5)}$$

In the formula, P denotes the average power of the pulsed laser ; τ is the pulse width of the pulsed laser ; f_p is the pulse frequency of the pulsed laser. During a pulse, the pulse period is

represented by a symbol T.

Equations (S1-S4) merely describe the Gaussian power density distribution of a stationary pulsed laser spot. To simulate the dynamic movement of the pulsed laser across the workpiece surface, the position coordinates of the laser center must be defined as a function of time. Since the developed finite element model is based on a two-dimensional orthogonal cutting geometry, the pulsed laser is simplified as a cross-sectional heat source acting on the ZX plane (where Z denotes the cutting direction and X denotes the depth of cut direction). Consequently, the effective radial distance R from the spot center can be expressed as:

$$R=\sqrt{(z-z_0)^2+(x-x_0)^2} \quad \text{(S6)}$$

In the formula, the point (z_0, x_0) is the center point of the pulsed laser. Since the pulsed laser moves on the Z-axis, z_0 and x_0 can be expressed as :

$$z_0=v_c nT \quad \text{(S7)}$$

$$x_0=0 \quad \text{(S8)}$$

In the formula, v_c is the moving speed of the laser, and n is the number of pulses acted by the instantaneous pulsed laser.

By arranging the formulas (S1-S5) to the formulas (S1-S8), the Gaussian distribution function of the pulsed laser power density moving on the z-axis is as follows :

$$I(r,z)=\begin{cases}\dfrac{P}{\pi\tau f_p r_b^2}\exp\left\{\dfrac{2\left[\sqrt{(z-v_c nT)^2+x^2}\right]^2}{r_b^2}\right\} & 0\le t\le\tau \\ 0 & \tau<t<T\end{cases} \quad \text{(S9)}$$

The temperature conduction model of pulsed laser assisted cutting is shown in figure S3. In the process of establishing the model, the heat transfer of the material should be considered at the same time. Because the established model is a two-dimensional geometric model, the temperature distribution of the workpiece in the two-dimensional coordinate system can be expressed as follows :

$$\frac{\partial}{\partial z}\left(k\frac{\partial T}{\partial z}\right)+\frac{\partial}{\partial x}\left(k\frac{\partial T}{\partial x}\right)+q_v=\rho c_p\frac{\partial T}{\partial t} \quad \text{(S10)}$$

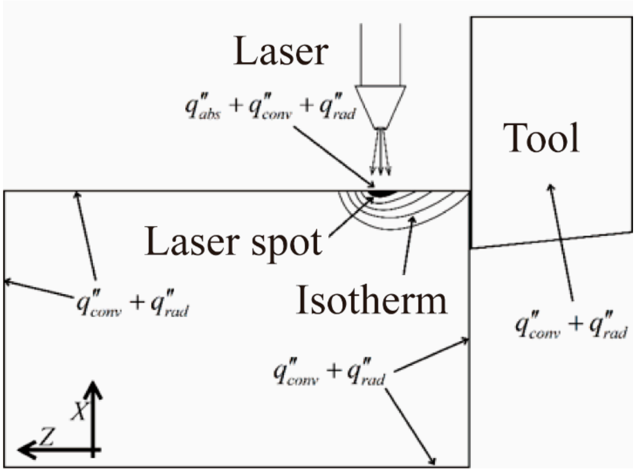

Figure S3 Temperature conduction model of pulsed laser assisted cutting

In the formula, the first two terms of the formula represent the energy diffusion in the Z direction and the X direction respectively, and the right side of the equation represents the increment of energy inside the workpiece. q_v (W / m^2) represents the power density of the internal heat source, ρ (kg / m^3) represents the density of the material, and C_P （J/kg·℃） represents the specific heat capacity of the material.

In Fig.S3 q''_{abs} (W / m^2) is the energy absorbed by the workpiece surface ; q''_{conv} (W / m^2) is the thermal convection energy on the surface of the workpiece ; q''_{rad} (W / m^2) is the thermal radiation energy of the workpiece surface. During the cutting process, both the workpiece and the tool will generate energy consumption due to convective heat transfer and thermal radiation. The convective heat transfer between the workpiece and the medium in the air can be expressed by Newton 's cooling formula :

$$q''_{conv} = h\Delta T \qquad \text{(S11)}$$

In the formula, h is the convective heat transfer coefficient, assuming that the convective heat transfer coefficient does not change with temperature.

The thermal radiation of the workpiece and the tool can be calculated by the Stefan-Boltzmann correction formula :

$$q''_{rad} = \sigma\varepsilon(T_1^4 - T_0^4) \qquad \text{(S12)}$$

where, σ is Stefan-Boltzmann constant, its value is 5.67×10^{-8} W/(m^2·°C^4); ε is the blackness ; T_1 is the temperature of the heated surface ; T_0 is the ambient temperature.

Before the tool does not contact the workpiece, the SiCp/Al composite material and the tool are in a normal temperature environment. Therefore, when the model is established, the temperature of the workpiece and the tool before cutting is considered to be normal temperature, where T_0 = 23 °C, and the boundary conditions can be expressed as :

$$T(z,x,t)_{t=0} = T_0 \qquad \text{(S13)}$$
